# Impacts of Early Life Stress on the Methylome and Transcriptome of Atlantic Salmon

**DOI:** 10.1038/s41598-017-05222-2

**Published:** 2017-07-10

**Authors:** Hooman K. Moghadam, Hanne Johnsen, Nicholas Robinson, Øivind Andersen, Even H. Jørgensen, Helge K. Johnsen, Vegar J. Bæhr, Helge Tveiten

**Affiliations:** 1 Nofima AS, Osloveien 1, NO-1433 Ås, Norway; 2 Nofima AS, Muninbakken 9-13, NO-9291 Tromsø, Norway; 3 Sustainable Aquaculture Laboratory - Temperate and Tropical (SALTT), School of BioSciences, The University of Melbourne, Parkville, Vic. 3010 Australia; 4 Department of Animal and Aquaculture Sciences, Norwegian University of Life Sciences (NMBU), NO-1430 Ås, Norway; 5 Department of Arctic & Marine Biology, University of Tromsø, NO-9037 Tromsø, Norway; 6 Norwegian College of Fishery Science, BFE, University of Tromsø, NO-9037 Tromsø, Norway

## Abstract

Exposure to environmental stressors during early-life stages can change the rate and timing of various developmental processes. Epigenetic marks affecting transcriptional regulation can be altered by such environmental stimuli. To assess how stress might affect the methylome and transcriptome in salmon, fish were treated using cold-shock and air-exposure from the eye-stage until start-feeding. The fish were either stressed prior to hatching (*E*), post-hatching (*PH*), pre- and post-hatching (*EPH*) or not stressed (*CO*). Assessing transcriptional abundances just prior to start feeding, *E* and *PH* individuals were found to have modified the expression of thousands of genes, many with important functions in developmental processes. The *EPH* individuals however, showed expression similar to those of *CO*, suggesting an adaptive response to extended periods of stress. The methylome of stressed individuals differed from that of the *CO*, suggesting the importance of environment in shaping methylation signatures. Through integration of methylation with transcription, we identified bases with potential regulatory functions, some 10s of kb away from the targeted genes. We then followed fish growth for an additional year. Individuals in *EPH* showed superior growth compared to other treatment groups, highlighting how stress can potentially have long-lasting effects on an organism’s ability to adapt to environmental perturbations.

## Introduction

In the face of a growing human population and changing environment, understanding the molecular basis of phenotypic differences between individuals is a fundamental requirement to ensure a robust and sustainable production of our farmed plants and animals. Over the past few decades, we have constantly optimized and improved methods for implementing efficient breeding programs, genotyping strategies and identifying variations associated with traits of interest. In recent years however, there also has been a growing appreciation for the importance of environmental factors and epigenetic mechanisms in regulating the expression of genes and contributing to phenotypic diversity^1, 2^. Based on the findings from human studies as well as a few model organisms, it is known that at least some environmental exposures, particularly those that are encountered during early life stages, can trigger developmental trajectories with lifetime impacts on the health, metabolism or behaviour of the animal^3^. Recent advances in genomics technologies, along with the availability of the reference genomes for many species of economic importance, now allow us to investigate the epigenetic mechanisms behind gene-by-environment interactions, with detailed precision at the molecular level across a broad range of organisms. For the production and management of farmed species, such knowledge will become increasingly more important, as it will provide a basis for utilizing environmental stimuli as a means to modulate the level of gene expression and subsequently influence the animal or plant’s physiology.

Atlantic salmon (*Salmo salar*), a member of the Salmonidae family, is a species of great social, cultural, environmental, evolutionary and economic importance with one of the highest market prices among all cultured fish^4^. As salmon aquaculture expands and becomes more resource intensive and industrialized, it needs to improve production efficiency without compromising animal welfare. Perhaps one of the most critical phases in Atlantic salmon farming is during the initial stages where the fish are transferred from freshwater to seawater^5^. Of the estimated 13–16% loss of total biomass during the seawater production cycle, the majority of mortalities and disease outbreaks occur in the first few months after seawater transfer^5–7^. These losses have mainly been attributed to multi-layers of stressors, including capturing, loading, transport, unloading and stocking. It has been suggested that such stressors can trigger a chain of molecular responses^8, 9^, which in return may supress the immune system^10^, seawater tolerance^11^, growth and/or survival of the fish^12, 13^. A recent report has traced back a large proportion of the seawater mortalities to certain hatcheries and suppliers, indicating that differences in hatchery practices may have significantly contributed to the physiological suitability of a fish to the challenges associated with the seawater transfer^7^.

DNA methylation at the CpG dinucleotides is one of the key epigenetic mechanisms that controls transcriptional expression and gene regulation^14^. It is well known that some environmental stimuli, particularly those encountered during early development, can trigger and contribute to variations in the methylation marks and patterns of gene expression^3, 15^. Such stimuli can potentially be used for conditioning fish to better cope with the various challenges that an animal faces in aquaculture settings. In this study, we aimed to investigate how different stress scenarios of cold shock and air exposure during early life stages in Atlantic salmon can influence the methylome landscape of the species and what are the subsequent impacts on the genome-wide regulation of gene expression. The stress treatments were adopted from a previous study in rainbow trout^16^ where it was shown that stress exposure during early development can reduce sensitivity to stress later in the juvenile fish. These types of stresses are relevant to the industry, as fish frequently encounter air exposure and experience changes in temperature during netting, sorting, treatments to remove ectoparasites and transportation (e.g., from 8 °C to 0 °C). We assessed and compared the growth performance of the fish from different stressed groups, up to 16 months of age, five weeks after the fish were transferred to the seawater. Using massively-parallel sequencing of mRNA and DNA, we found genomic sites from different stress groups to have vastly altered their methylation signatures compared to those of the unstressed individuals. Through integration of methylation data with gene expression information and comparative assessment of the omics profiles across different groups, we identified putatively important regulatory sites, that apparently play key roles in controlling the level of transcript expression. While we identified thousands of genes that had significantly modified their expression magnitude in response to stress, animals that had received stress treatment over more extended periods of time showed an unexpected convergence of their gene expression patterns to those of the unstressed individuals, an evidence suggestive of the animal’s adaptive response to stress. Interestingly these fish also showed a superior growth performance compared to the fish from other treatment groups. We suggest that this improved performance might be due to the biological concept of “hormesis”, the findings that low or moderate exposure to otherwise harmful stimuli can protect or improve one’s tolerance to the same (or other) stimuli/stressor during later stages of life^17^.

## Results and Discussion

### Methodology overview

Following fertilization, embryos from a full-sib family were divided into four triplicate groups of *i*. unstressed control (*CO*), *ii*. stressed during embryogenesis (*E*), *iii*. stressed during post-hatch (*PH*), and *iv*. stressed during both embryonic and post-hatch stages (*EPH*). The stress treatment was in the form of a cold-shock (drop from 7 °C to 0.2 °C for 1 min) that was followed by air exposure (15 °C) for 1 min before the fish were transferred back to a 7 °C water. Just prior to the start feeding (880 d°), 6 individuals per group were randomly selected and genomic DNA and total RNA were extracted from the same individuals (whole fry) for further analyses using RNA and Reduced Representation Bisulfite Sequencing (RRBS; Methyl-MiniSeq) on an Illumia platform.

The genomic and transcriptomic short read sequences were mapped to the Atlantic salmon assembly ICSASG_v2. The gene expression profiles were assessed using the Tuxedo pipeline^18^ and the methylation patterns were investigated using Bismark^19^ along with the R package, methylKit^20^. After start feeding, the stress treatments were ceased and all groups were maintained under standard hatchery conditions in freshwater until smoltification. The fish were then transferred to the seawater where a 35-day growth trial was undertaken. The trial ended almost one year after the last stress exposure. Detailed experimental descriptions can be found in the Materials and Methods.

### Stress induced differences in transcriptome profiles

Due to the pseudo-tetraploid nature of the Atlantic salmon genome^21, 22^ and to obtain a more accurate overview of the gene expression profiles, transcriptomes were sequenced to a great depth. On average, 60 M reads per individual were obtained and 93% were successfully mapped to the Atlantic salmon genome (Supplementary Figure 1a,b). Transcripts corresponding to 40,159 genes were detected, of which 67% and 98% showed significant hits against the NCBI non-redundant protein (nr) and nucleotide (nt) databases respectively. The predicted proteins exhibit high diversity of functional properties, including immune response, lipid metabolism, methyltransferase activity and oxidoreductase activity (138, 102, 178 and 259 genes respectively, Supplementary Figure 2).

**Figure 1:**
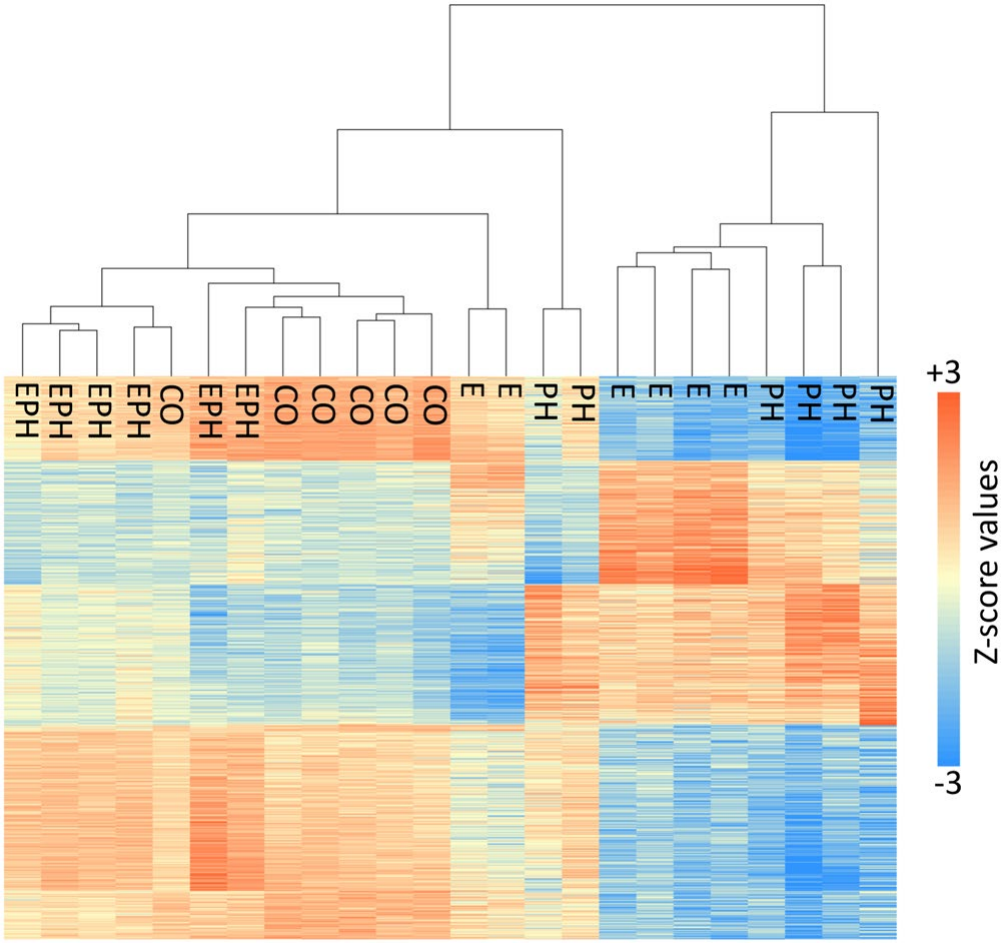
Heatmap of the standardized FPKM gene expression, showing relative levels of abundance of transcripts across the control (*CO*), embryonic (*E*), post-hatch (*PH*) and embryonic and post-hatch (*EPH*) treatment individuals for genes with greater than 1.5 fold-change difference in expression.

The principal component analysis (PCA) of the gene expression data along with the clustering of transcripts with fold-change differences in expression greater than 1.5, further showed variations in gene expression among individuals within the *E* and *PH* treatment groups (Fig. 1 and Supplementary Figures 3a and 4). In particular, two individuals within each group, had expressions partly similar to the *CO* individuals and partly similar to the rest of the individuals in the *E* and *PH* groups. It is possible that many of the genes in these individuals have started to revert to the expression levels found under the “pre-stress” conditions. The underlying causes of such variation can be due to different biological or environmental factors including genotype, *G* × *E* interactions, epistasis, gender, potential differences in developmental stage and heterogeneity of the experimental setup. Such differences are an inherent part of many complex biological systems and reflect the plasticity of gene regulation and expression in response to environmental stimuli.

**Figure 2:**
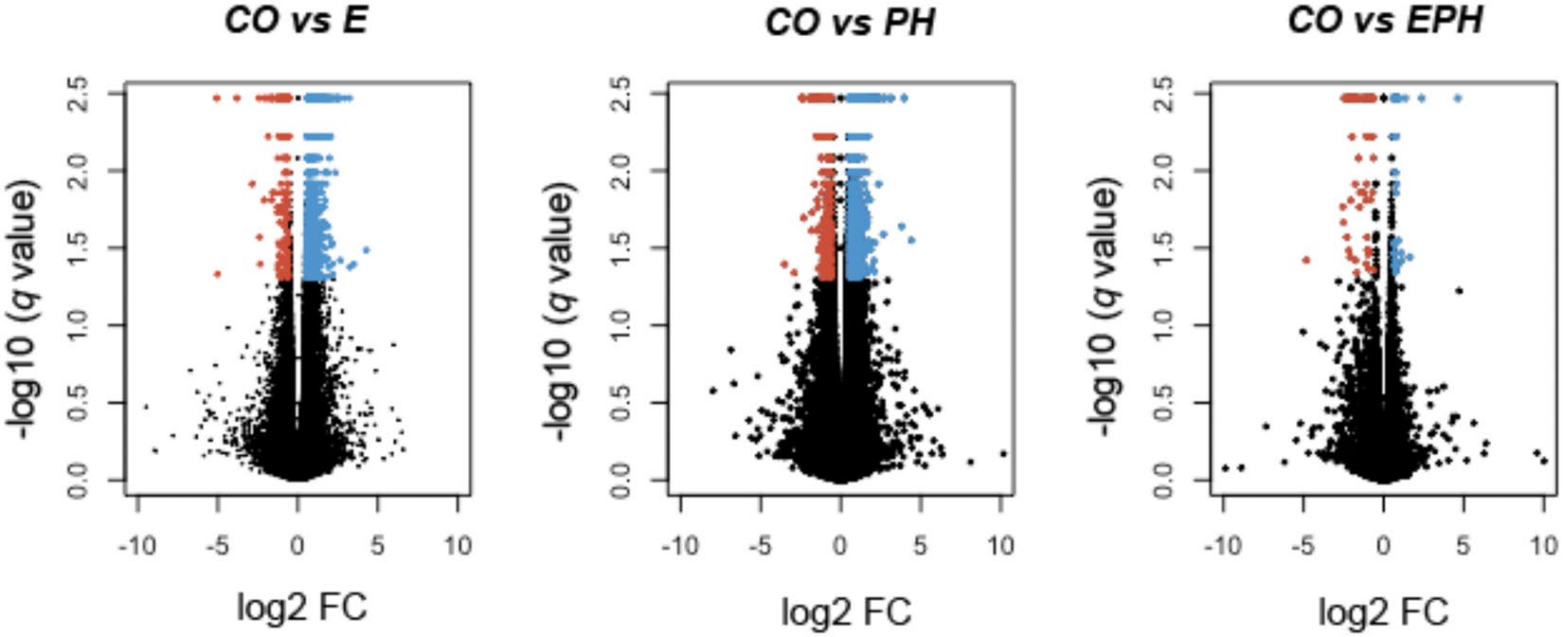
Volcano plots, showing genes with biased patterns of expression between individuals in the control group (*CO*) and the fish stressed during embryonic (*E*), post-hatch (*PH*) and embryonic and post-hatch (*EPH*) stages. Red dots represent genes with significantly lower abundances while the blue dots show genes with higher levels of expression. Values on the x- and the y-axes are log_2_ fold change (FC) differences in the gene expression and negative log_10_ of the corrected *p* values respectively.

Comparison of transcript abundances for individuals who received stress during *E* or *PH* stages to those of the unstressed individuals showed that 1,776 and 2,700 genes were differentially expressed in the *E* and the *PH* experimental groups respectively (Fig. 2 and Supplementary Figure 4). Most of the differentially expressed genes showed higher levels of expression among the stressed individuals. For the *E* group comparison, 1,534 genes had higher transcript abundances while only 242 had lower expression. For the *PH* group, the numbers of biased genes with higher or lower transcript abundances were 2,206 and 494, respectively. The genes with highest fold-change differences in both stress comparisons included: *i*. Thymidine phosphorylase, a gene that plays a key role in angiogenesis and is usually strongly up-regulated in tumour cells^23^; *ii*. Transforming growth factor beta (*TGF*-beta) receptor type II, with important functions in signal transduction, affecting cell proliferation and differentiation^24^ and is associated with the development of various types of tumours^25^; *iii*. Mucin-12-like product, a member of the mucin gene family which is involved in epithelial cell protection, signalling and growth regulation^26^ and found to be differentially expressed in various cancers and other diseases e.g., refs 27–30; and *iv*. Apolipoprotein B mRNA editing enzyme, a C to U editing enzyme which plays a role in the epigenetic regulation of gene expression through DNA demethylation^31^ (Supplementary Figure 5).

Enrichment analysis of gene ontology (GO) terms found biological processes associated with cell movement and cellular organization to be among the top over-represented functional categories in both *E* and *PH* stressed individuals (Supplementary Figure 6a). These are necessary steps for processes such as embryonic morphogenesis, angiogenesis, immune system development, tissue repair and regeneration^32–35^, all mainly relying on the dynamics of actin assembly^36^ (Supplementary Figure 6a). Disturbance in the regulation and function of actin and other cytoskeletal components is one of the main underlying causes of a wide range of diseases and abnormalities, from cancer to muscle skeletal disorders^34, 37, 38^. Our data suggest that stress during early stages of salmon development can modify the transcription and regulation profiles of a number of genes that are involved in the proper functioning of these networks.

Enrichment analysis also detected under-representation of GO functional annotations associated with translation and protein synthesis (Supplementary Figure 6b). Many of the genes associated with this term act at the ribosomal and translation initiation levels, suggesting that stressed cells might be compensating for high expression levels of genes such as those that are involved in cell differentiation and proliferation, through curbing their translation. Significantly lower expression levels of genes involved in vision development and phototransduction, including several opsins and RPE-retinal G protein-coupled receptors (Supplementary Figure 6b), were also detected among the *E* and *PH* individuals compared to the unstressed individuals. Genes encoding opsins, determining sensitivity for red (two different gene copies) and green light, as well as duplicated copies of RPE-retinal G protein-coupled receptor, all showed reduction in their expression within these two stressed categories. Heat and cold stress have been shown to repress the transcriptional expression of genes involved in phototransduction in zebrafish^39, 40^. These findings highlight the likely involvement of environmental cues such as sub-optimal temperatures in the development of phototransduction and visual regulatory processes.

We also investigated the functional properties of the genes that were differentially expressed between the *E* and the *PH* stressed groups. In total, 714 genes were identified, with 406 and 308 genes having higher levels of expression in the *E* and the *PH* individuals respectively. Many of the genes having higher expression in the *PH* group had functions associated with the regulation of cell growth. Some of these genes included different members of the insulin-like growth factor binding protein family (*IGFBP-1*, *IGFBP-6*, *IGFBP-1B2*), implicated in modulating various bioactivities of the IGF system^41^ and connective tissue growth factor (*CTGF*), a protein important in different cellular activities, including angiogenesis and skeletogenesis^42–44^. Interestingly, within this stressed group of individuals, we also found up-regulation of different members of the growth arrest and DNA damage-inducible protein gene family (*GADD45-alpha*, *-beta* and *-gamma*). These proteins are suggested as “stress sensors”, all involved in growth suppression, cell-cycle arrest and apoptosis^45–47^. Lower levels of expression of different immune response components were also detected in the *PH* individuals, including various members of the chemokine (C-C motif) ligand, *CCL4*, *CCL19*, *CCL20* and *CCL28* all playing important roles in both innate and adaptive immunity^48–50^.

Unlike the *E* and *PH* stressed individuals, the transcriptional signatures of the *EPH* replicates showed very high resemblance to the gene expression patterns of the unstressed group (Figs 1 and 2 and Supplementary Figures 3a and 4). Only 108 genes were differentially expressed, with 69 having lower and 39 showing higher gene expression levels. Nonetheless, the overall profiles of the transcripts and the general direction of changes in the expression showed high consistency across all stressed groups (Supplementary Figure 7a,b). Among differentially expressed genes within *EPH*, and similar to the other stressed groups, the *TGF*-beta receptor type II showed the highest increase in expression with approximately 25-fold increase in the abundance of its transcripts (Supplementary Figure 5b). We also identified a unique expression signature for the metastasis suppressor protein 1-like gene (*MTSS1*), which was only expressed in the *EPH* treatment group. *MTSS1* is known for its ability to suppress cell metastasis and provide a means to regulate cell proliferation, and also acts as a cytoskeletal scaffold to regulate actin dynamics^51^. This is consistent with the results from functional enrichment assessment obtained from the *E* and *PH* treatment fish, in that temperature and possibly some other environmental stressors can modify the regulation of cell proliferation and the process of actin assembly^52^. The increased transcription of *MTSS1* could also function to mitigate the significantly reduced expression of two different MHC class I genes, as observed among the *EPH* individuals. It is now well established that the down-regulation of MHC class I genes is tightly associate to cell metastasis, as this process can greatly facilitate the concealment of the tumour cells from T cell-mediated immune response^53, 54^.

Despite these differentially expressed genes, the overall genome-wide profiles of transcription between *EPH* and *CO* individuals seem to have converged to a great extent (Figs 1 and 2 and Supplementary Figures 3a and 4). Our data provide support that a wide range of transcriptomic reactions can be triggered during the initial phases of stress. However, over time, individuals will become more adapted and can cope more efficiently with such environmental stimuli. Within the *EPH* group, the transcriptomic signatures of such an adaptation are reflected in the expression patterns of thousands of genes that have modified their transcription levels, making them comparable to the “pre-stress” state.

Based on our expressed sequence information, we also identified signatures of many duplicated genes and conserved blocks of syntenic paralogous regions between different chromosomes (Supplementary Figure 8). Using information from the uniquely aligned reads, we further investigated the profiles of gene duplicates where the expressions of both copies were significantly modified in response to stress. We found that in cases where the two paralogues have significantly changed their expression patterns compared to the control, the direction of the change in expression was the same between the two copies, such that both duplicates exhibited either higher or lower abundances in transcriptional levels (Supplementary Figure 9). Some genes of interest, such as actin muscle, collagen alpha 1, heat shock protein beta 1, lipopolysaccharide-induced tumour necrosis factor-alpha, hemoglobin embryonic subunit alpha, nephronectin and different members of the myosin family also showed consistently higher expression of their two paralogues in both *E* and the *PH* groups (Supplementary Figure 10). However, further research is needed to determine whether the parallel changes in expression for these paralogues is due to the conserved regulatory control of the same genetic mechanisms or whether different regulatory elements have responded to stress in a convergent manner.

### Stress induced differences in methylome profiles

We next investigated the genome-wide impacts of stress on the methylome signatures across the different stressed groups. On average, about 34 million paired-end reads per individual were sequenced with RRBS and more than 80% of these reads were successfully mapped to the salmon genome (Supplementary Figure 1b). Analyses of the differentially methylated dinucleotides (DM), obtained through pairwise comparisons with the *CO* group, identified, on average, 16% more sites exhibiting hyper-methylation than hypo-methylation among the stressed individuals (Supplementary Figure 11). Altogether, we identified 3,137 hyper-methylated and 2,458 hypo-methylated regions across all groups. The percentage of the globally methylated Cs in the CpG context ranged from 77–80%, with the highest percentage of methylation observed among the *EPH* individuals (Supplementary Figure 12).

**Figure 3:**
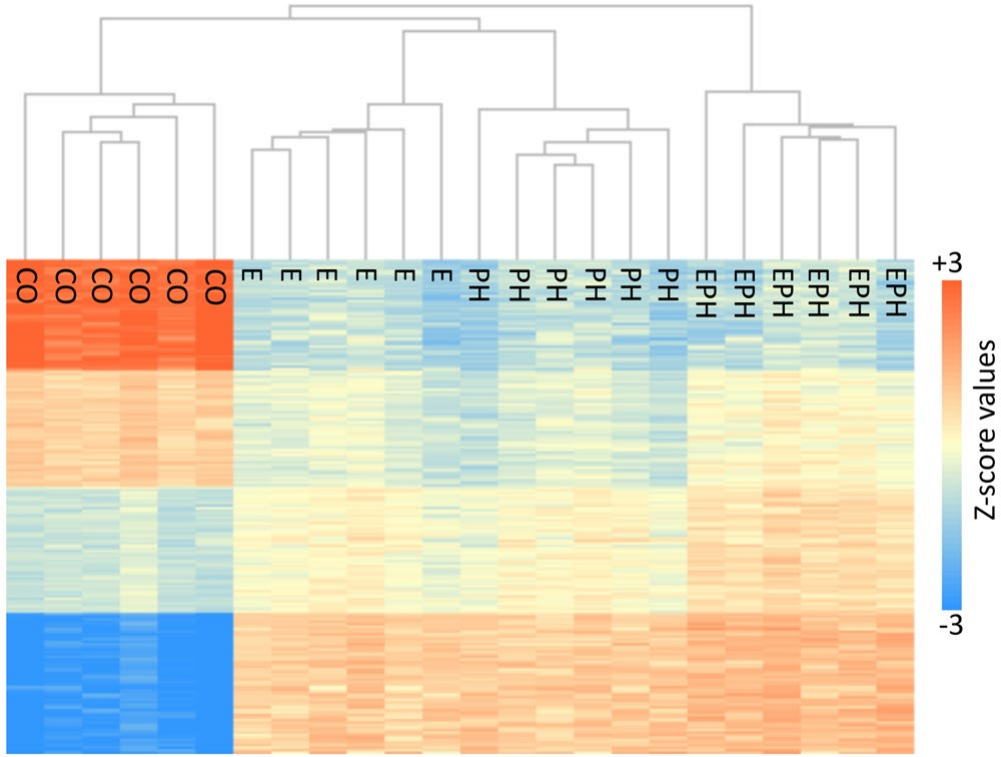
Heatmap, showing the standardized methylation ratios of the DM CpG dinucleotides, identified between the control group (*CO*) with individuals in the stressed embryonic (*E*), post-hatch (*PH*) and embryonic and post-hatch (*EPH*) groups. Each horizontal line represents a distinct DM site.

Similar to the findings in gene expression, we also identified a very high concordance in the directional changes of DM sites among stressed individuals relative to the *CO* group (Supplementary Figure 7c,d). These findings are important, as through the converged patterns of methylation data we can more confidently localise the genomic positions of CpG dinucleotides that have responded to the stress stimuli through the gain or loss of methyl groups. The majority of these DM loci are located within intergenic regions (49%), with fewer numbers in putative promoters (6%), 5′ UTR (4%), intronic (33%), exonic (5%) and 3′ UTR (3%) regions. Clustering of the DM loci in addition to the PCA plot also showed a clear pattern of genome-wide changes across methylation sites within different stressed groups compared to control individuals (Fig. 3 and Supplementary Figure 3b). Interestingly, there was a high concordance in the clustering profiles of the loci that were found to be DM in the *E* and the *PH* experimental groups (Fig. 3 and Supplementary Figure 3b), even though these individuals received stress during different ontological stages. On the other hand, the methylation heatmap of the *EPH* individuals is distinctly different to the *E* or *PH* groups. These findings suggest that, at least in our experiment, the duration, rather than the ontological stage at which an individual received stress, played a more critical role in determining the profile of the methylome. However, the generality of these findings needs further confirmation. Further, while by using the whole fry, we ensured that all tissues are included in the analyses, our data is limited in identifying tissue specific context in response to stress, for both expression and methylation.

We next examined the association between changes in DNA methylation with the abundance of transcription in a subset of genes that showed consistently higher or lower levels of expression (minimum 1.5 fold change difference in transcript abundances) across all stressed groups compared to the control. Among 108 biased genes investigated (and their 10 kb flanking regions), significant changes in the average methylation gain or loss was detected (Welch two sample t-test; *p* < 0.0001). Of these genes, 45% showed increased levels of methylation with significant reduction in their expression magnitude (Supplementary Figure 13a). These hyper-methylated genes included methyltransferase-like protein 25, actin alpha cardiac muscle l, actin beta, guanidinoacetate N-methyltransferase, death-associated protein, tyrosine phosphatase type IVA2, tyrosine-phosphatase receptor type G, serine/threonine-phosphatase 2 A and pancreatic progenitor cell differentiation and proliferation, all playing an important role in cell cycle control and in the regulation of cell growth, proliferation and differentiation e.g., refs 55–58 (Supplementary Table 1). Suggestive evidence of hyper-methylated sites associated with increased transcript expression as well as hypo-methylated sites associated with either higher or lower levels of expression were also identified in 25%, 16% and 14% of the cases investigated respectively (Supplementary Figure 13b,c,d). These categories contained multiple members of particular gene families, further confirming the functional properties that might be the targets of regulation via methylation mechanisms. For instance, different members of methyltransferase, myosin, actin, collagen and protein phosphatase gene families showed different and mostly complementary patterns of regulation and methylation (Supplementary Table 1). Alternative transcriptional profiles among different members of the same gene family might indicate compensatory functional mechanisms between these genes.

**Figure 4:**
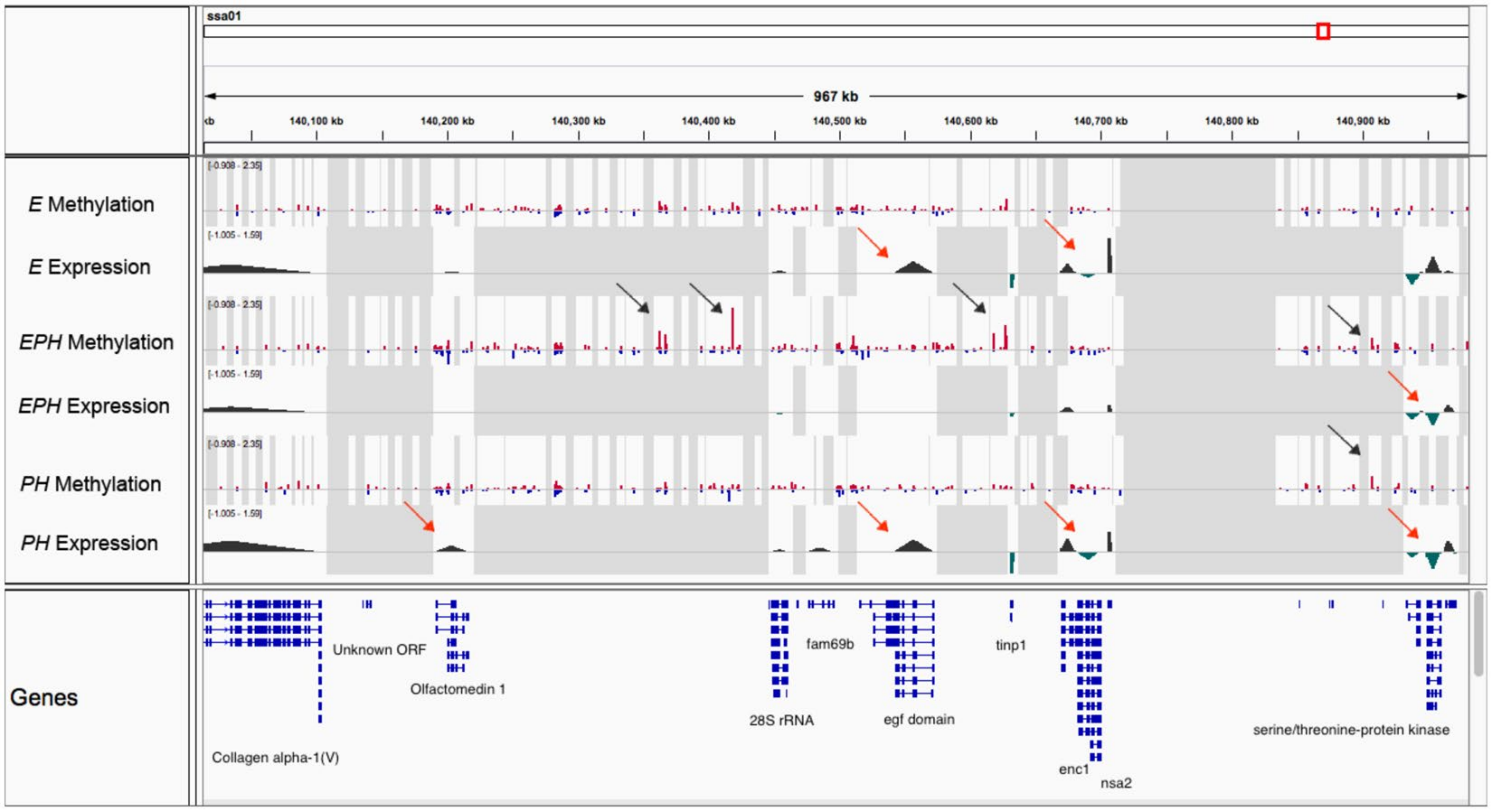
An example of a genomic bin on chromosome 1, showing suggestive association between differentially methylated sites with differentially expressed genes. The red and the blue bars show higher or lower levels of methylation relative to the control individuals in the stressed embryonic (*E*), post-hatch (*PH*) and embryonic and post-hatch (*EPH*) groups. The bars indicated by dark arrows are significantly hyper-methylated regions. The green peaks and valleys are log_2_ fold-change differences in gene expression. The red arrows show genes that have been regulated differently compared to the un-stressed individuals.

To identify CpG sites with putatively important regulatory impact on gene transcription, we scanned sliding windows of 1 Mb genomic bins throughout the genome. We detected potentially key regulatory loci, some 10s to 100s of kb up- or down-stream of the differentially expressed genes. An example of one such genomic window, identified on chromosome 1, is presented in Fig. 4. As depicted in the figure, a number of genes including epidermal growth factor (*EGF*) domain and ectodermal-neural cortex 1 (*ENC*1) show significant differences in their expression profiles in the *E* and *PH* groups compared to the control group. Little evidence of any DM loci within this genomic block was found across both of these treatment groups. On the other hand, within the *EPH* individuals, while the expression of the majority of the genes identified in this region exhibit a similar pattern to those of the unstressed individuals, we also identified a number of CpG sites with significant increase in their levels of methylation. Taken together and comparing the transcription and methylation profiles across the three treatment groups, it can be speculated that the increased methylation in this region had functioned to re-adjust the level of transcription to the “pre-stress” state within the *EPH* individuals.

### Comparative growth performance and evidence of stress-related benefits on growth

**Figure 5:**
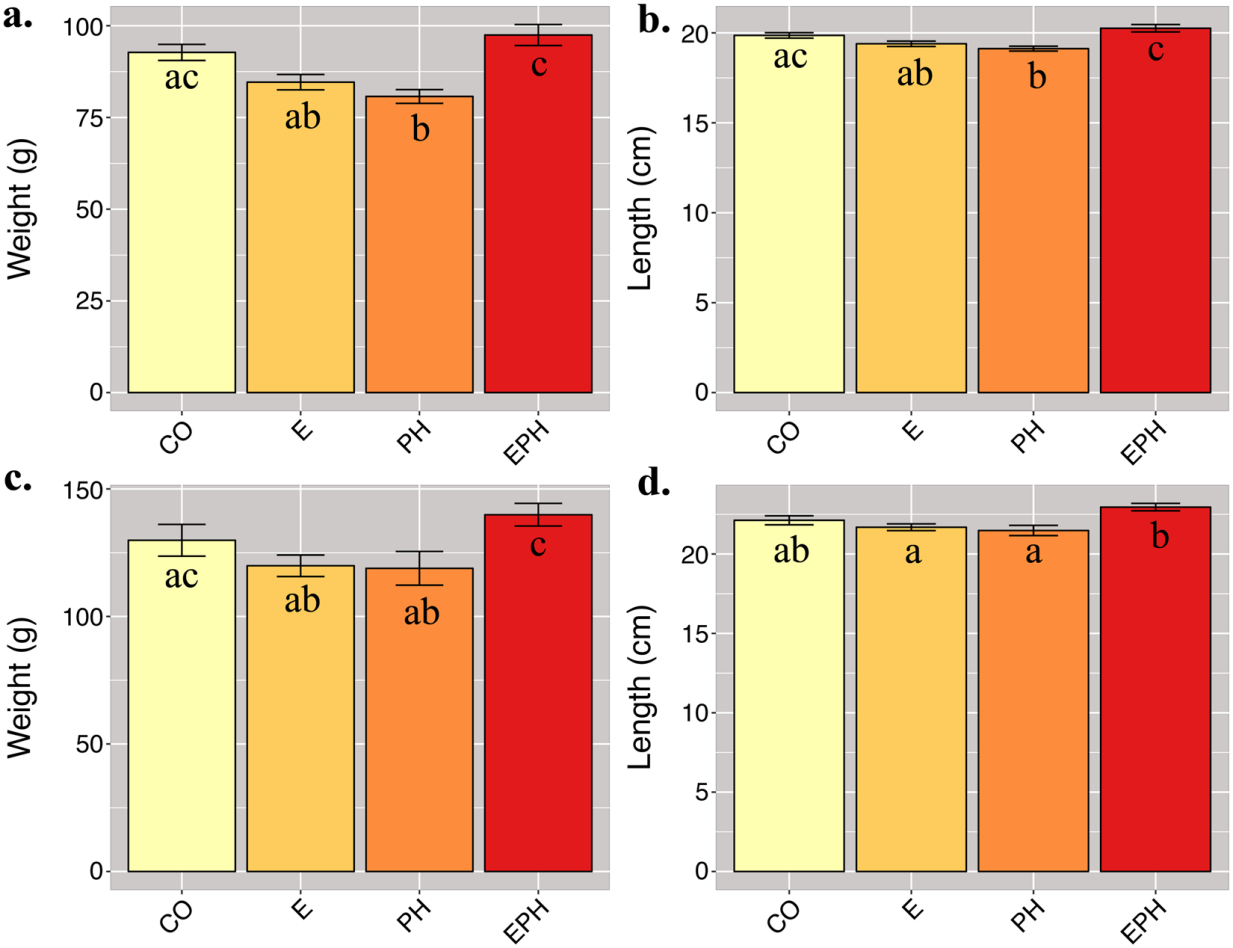
Weight (g) (**a** and **c**) and length (cm) (**b** and **d**) of the fish in different treatment groups at the time of transfer to the seawater (day 0) (**a** and **b**) or 35 days after seawater transfer (35‰ salinity) (**c** and **d**). Different superscript letters indicate statistical significant differences (*p* < 0.05).

To assess the potential influence of stress on growth performance during early life stages, 30 individuals from each group were measured for their body weight just prior to the start feeding. No significant, adverse effect of stress was identified on the animal growth (data not shown). From the start feeding on, all fish were treated and raised under the same standard hatchery conditions and were monitored for their growth performance. Fish within the *EPH* group were found to be significantly larger by weight (~16%) (*p* < 0.033) and by length (~5%) (*p* < 0.003) than fish in the other stress treatment groups. The better growth performance was maintained during both freshwater and seawater stages (Fig. 5). Further, although not statistically significant, the *EPH* fish were also heavier (~6%) and longer (~3%) than the fish in the control group. This could suggest that the *EPH* fish were better able to handle subsequent stresses at later stages of life, and as a result, more efficiently allocated energy and metabolic resources towards improving their growth and development. These findings can best be explained by the phenomenon of hormesis, an adaptive response of a cell or an organism to low or moderate levels of stress, where after an initial disruption to homeostasis it can act to modify the production levels of beneficial and/or harmful gene products^59^. If this has been the case in the fish examined in this study, these effects have probably been maintained for at least one year after the stress treatments were ceased. Although currently little is known about the molecular basis of hormetic mechanisms^17^, the positive effects of mild, repeated stress have been documented in many species e.g., refs 60–62. For example, fruit flies subjected to a mild stress, such as cold, substantially increased the resistance to severe stresses like heat or fungal infections, and additionally, these effects seem to persist throughout the life of the animal^63^. In the light of these findings, our study provides insight into the gene products and genome-wide regulatory sites that are sensitive to methylation modifications and might be important for conferring better growth performance in the farmed Atlantic salmon.

## Materials and Methods

### Sample collection and preparation

Batches of milt and unfertilized salmon eggs were obtained from a commercial supplier (AquaGen, Trondheim, Norway). Fertilized eggs and alevins prior to exogenous feeding are exempt from the Norwegian Regulation on Animal Experimentation, and thus approval of the experimental protocol for this experiment by the Norwegian Animal Research Authority (NARA) was not required. However, all experiments and trials were conducted in accordance with the laws and regulations controlling experiments and procedures in live animals in Norway (the Animal Welfare Act of December 20th 1974, No 73, chapter VI sections 20–22 and the Regulation on Animal Experimentation of January 15th 1996). Fertilization was performed according to standard procedures using milt in excess at the Nofima’s Aquaculture research station (Tromsø, Norway). Egg/embryo incubation was performed in upwelling incubators at a water temperature of 7 °C. Eggs from a full-sib family were divided into four groups: *i*. unstressed control (*CO*), *ii*. eggs stressed during embryogenesis (*E*), *iii*. fry stressed during post-hatch stages (*PH*), *iv*. embryos and post-hatch fry stressed during both during embryonic and post-hatch stages (*EPH*). Each treatment group was incubated in triplicate trays containing ca. 1800 eggs in each replicate. The different treatment groups were subjected to bouts of stress (exposure to cold water (0.2 °C) for 1 min, followed by air exposure (15 °C) for 1 min and then return to 7 °C water) either during embryogenesis (5 times from 250 to 450 day degrees, d°) or during the yolk-sac stages (3 times, from 540 to 800 d°), or both. The time point just prior to start feeding (880 d°) was chosen for further analyses using RNA and Reduced Representation Bisulfite Sequencing (RRBS; Methyl-MiniSeq). Samples were secured by immersion in RNA-later (Ambion) according to the manufacturers protocol. Six individuals from each group were randomly chosen (total n = 24). Total RNA and genomic DNA were extracted from the same individuals (whole fry) using the AllPrep DNA/RNA/miRNA Universal Kit (Qiagen). Samples were shipped to the Zymo Research (San Diego, CA, USA) on dry ice and arrived in good condition as confirmed by Bioanalyzer (Agilent).

### RNA sequencing, alignment and differential expression assessment

Preparation of the mRNA libraries and sequencing transcripts were performed by the Zymo Research (San Diego, CA, USA) using standard protocols (www.illumina.com). Samples were sequenced on an Illumina HiSeq platform as single-end 50 bp reads. Following read quality assessment (www.bioinformatics.babraham.ac.uk/projects/fastqc/), removing sequencing adapters and trimming low quality bases^64^, the remaining sequences were aligned to the salmon genome assembly ICSASG_v2 using TopHat (v.2.0.13)^65^. To obtain a more comprehensive view of the expressed genes and transcripts, we further included annotation information from previously processed RNA sequence data, from an additional 494 individuals, representing different populations, different developmental stages and obtained from tissues such as fillet muscle, heart muscle, liver and whole embryo (unpublished data). The aligned sequences where then fed to Cufflinks^18, 66^ to generate transcriptome assemblies for each sequenced sample and merged together by Cuffmerge to construct a uniform gene transfer file. Expression data were normalized via the median of the geometric means of fragment counts across all sample^67^. Cuffdiff was used to estimate the expression abundances of the assembled genes and transcripts and to test for differential levels of expression between all stressed groups. Considering the duplicated origin of the Atlantic salmon genome, differential expression assessments were preformed once using the information from only the uniquely aligned reads and once using all the mapped sequences. The results were almost identical between the two datasets, for both single and duplicated gene copies. Genes or transcripts with greater than 1.5-fold difference in expression and corrected *p*-values of less than 0.05 were assigned as differentially expressed.

### RRBS sequencing (Methyl-MiniSeq), alignment and differential methylation assessment

Libraries were prepared from 200–500 ng of gDNA digested using 60 units of *TaqαI* and 30 units of *MspI* (New England Biolabs, Ipswich, MA, USA) and then extracted with Zymo Research DNA Clean & Concentrator-5 kit (ZR). Fragments were ligated to pre-annealed adapters containing 5′-methyl-cytosine instead of cytosine according to the manufacturers guidelines (Illumina Inc., San Diego, CA, USA). Adaptor-ligated fragments of 150–250 bp and 250–350 bp in size were recovered from a 2.5% NuSieve 1:1 agarose gel (Zymoclean Gel DNA Recovery Kit, ZR). The fragments were then bisulfite-treated using the EZ DNA Methylation-Lightning Kit (ZR). Preparative-scale PCR was performed and the resulting products were purified (DNA Clean & Concentrator, ZR) and sequenced on an Illumina HiSeq genome analyzer as paired-end, 50 bp fragments.

Following the initial assessment of sequence quality using FastQC (http://www.bioinformatics.babraham.ac.uk/projects/fastqc/), sequencing adapters and low quality bases were removed (www.bioinformatics.babraham.ac.uk/projects/trim_galore/) ref. 64 to allow only the highest quality sequences to be used in subsequent analyses. Bisulfite treated reads were then mapped to the Atlantic salmon reference assembly ICSASG_v2 using Bismark v0.12.3^19^ with Bowtie1^68, 69^. The methylation status of the CpG dinucleotides was extracted from the uniquely aligned reads using the Bismark methylation extractor module. The R package, methylKit^20^ was used to identify differentially methylated bases by implementing a logistic regression test. The *p*-values of the methylation tests were corrected for multiple testing (i.e., *q*-value) using a sliding linear model suitable for datasets with dependence structures^70^. A CpG site was assigned to have a differential methylation pattern when absolute methylation difference was greater than 20% and *q*-value less than 0.1. All raw sequences have been deposited to the NCBI Short Read Archive (SRA) under the BioProject ID PRJNA388534.

### Gene annotation and enrichment assessment of Gene Ontology terms

Putative open reading frames were identified using TransDecoder^71^. Functional annotations of the Gene Ontology (GO) terms were assigned using Blast2GO^72^ against the SwissProt/TrEMBL and the NCBI nr databases and further mapped to more generic terms by GO-Slim (www.geneontology.org/GO.slims.shtml). Enrichment in GO terms for genes with biased pattern of expression between different groups were examined by the R package topGO^73^ and the significance level was assessed using Fisher’s exact test.

### Identification of expressed, duplicated genomic regions

To identify duplicated genomic blocks, the longest transcript from each gene was extracted and used as a source to create a database of non-redundant, expressed sequences. Nucleotide blast search was then used as a mean to compare this database against itself using a cut-off e-value of 10^−10^. Duplicated genomic blocks were assigned between any two independent genomic regions if at least 10 neighboring genes with the second best reciprocal blast hit were identified between these blocks.

### Associating methylation with gene expression

To investigate putative associations between transcript abundances with average changes in DNA methylation, a subset of genes with consistent patterns of differential expression among all stressed groups were selected (Supplemental Table 1). After excluding CpG dinucleotides with minimum average coverage of less than 6 reads per sample as well as sites with cut-off value of less than 8% difference in methylation gain or loss, the mean methylation of the gene body and the flanking 10 Kb up- and down-stream regions of the genes were calculated. Statistical differences in the average methylation changes were tested for all pairwise comparisons using Welch’s two samples t-test. Patterns of changes in methylation and gene expression were also investigated in 1 Mb sliding windows. In particular, genomic blocks containing differentially expressed genes were examined more closely to identify putatively regulatory sites that are sensitive to methylation modification.

### Comparative assessment of growth performance

In order to evaluate any further influence of stress on long-term development (here growth rate), 880 d° fry from all treatment groups were moved to start feeding units and fed in duplicate tanks under standard hatchery conditions (continuous light and 8 °C water, gradually increasing to 10 °C over two weeks). All groups were maintained under these conditions (in fresh water) for about seven months until they were c. 45 g. Smoltification was induced using a standard hatchery protocol. Briefly, the fish was transferred from continuous light and 10 °C water temperature to a 6 week long “winter” period with short day length (8 h light/16 h darkness; 8 L:16D) and 6 °C, after which they were transferred to a “summer” period with continuous light and 10 °C. Smoltification was completed 6 weeks later. In mid-March 2014 all groups had developed full osmoregulatory capacity when exposed to seawater (35‰ salinity). Sub-populations of the different treatment groups were subjected to a seawater challenge test (exposure to full strength seawater (35‰ salinity) at 7 °C for 24 h). Normal plasma chloride concentration (c. 140 mM) and plasma osmolality (c. 340 mOsmol) after 24 h is indicative of full hypoosmoregulatory capacity. Fish from different treatment groups were transferred to seawater and a 35-day growth trail was undertaken. This growth trail ended almost exactly (within a couple of days) one year after the last stress exposure.

## Electronic supplementary material

Supplementary Data
