# Supplementary material for: Impacts of Early Life Stress on the Methylome and Transcriptome of Atlantic Salmon
Source: Sci Rep. 2017 Jul 10;7:5023. doi: 10.1038/s41598-017-05222-2 (PMC5504078; doi:10.1038/s41598-017-05222-2)
Supplement: Supplementary file 1 — Supplementary Data [file 41598_2017_5222_MOESM1_ESM.pdf]

IMPACTS OF EARLY LIFE STRESS ON THE METHYLOME AND TRANSCRIPTOME OF ATLANTIC SALMON

Hooman K. Moghadam[1], Hanne Johnsen[2], Nicholas Robinson[1,3], Øivind Andersen[1,4], Even Jørgensen[5], Helge K. Johnsen[5], Vegar J. Bæhr[5] and Helge Tveiten[2]

[1]*Nofima AS, Osloveien 1, NO-1433 Ås, Norway*;

[2]*Nofima AS, Muninbakken 9-13, NO-9291 Tromsø, Norway*;

[3]*Sustainable Aquaculture Laboratory - Temperate and Tropical (SALTT), School of BioSciences, The University of Melbourne, Parkville, Vic. 3010, Australia*

[4]*Department of Animal and Aquaculture Sciences, Norwegian University of Life Sciences (NMBU), NO-1430 Ås, Norway*;

[5]*Department of Arctic & Marine Biology, University of Tromsø, NO-9037 Tromsø, Norway*;

Article type: Research paper

Running title: Stress, methylation and transcription in Atlantic salmon

Corresponding author:

Hooman K. Moghadam: hooman.moghadam@nofima.no

Supplementary Figures

Supplementary Figure 1. Bar chart depicting the total number of sequenced reads (green), the total number of mapped reads (yellow) and the total number of uniquely mapped reads (black) per individual in RNA-Seq (a) and RRBS (b) analyses.

Supplementary Figure 2. Bar graphs showing the top functional categories in the three gene ontology domains, biological process, molecular function and cellular component. The numbers on the x axes represent the number of genes within each functional class.

Supplementary Figure 3. Principal component analysis (PCA) of gene expression (a) and methylation (b) profiles across unstressed individuals (*CO*) and individuals that received stress during embryonic (*E*), post-hatch (*PH*) and embryonic and post-hatch (*EPH*) stages.

Supplementary Figure 4. Scatter plot of the log transformed FPKM expression data between the stressed groups with the control individuals. The blue dots show genes with elevated levels of expression while the red dots are genes with lower expression abundances compared to the control.

Supplementary Figure 5. Graphs showing the mean ± SEM of the FPKM expression units for the control (*CO*) as well as the stressed treatment groups for (a) thymidine phosphorylase, (b) TGF beta receptor 2, (c) mucin 12-like gene and (d) apolipoprotein B mRNA editing enzyme, catalytic polypeptide-like 2.

Supplementary Figure 6. Significantly over-represented (a) and under-represented (b) biological processes of differentially expressed genes between the embryonic (*E*) and post-hatch (*PH*) stress treatment groups with the unstressed individuals.

Supplementary Figure 7. Venn diagrams showing consistent patterns in change of gene expression (a and b) and methylation (c and d) in response to stress across the three treatment groups, embryonic (*E*), post-hatch (*PH*) and embryonic and post-hatch (*EPH*). The figures show if a gene was significantly found have higher (a) or lower (d) expression in at least one treatment group, that gene most likely had the same pattern of change in its expression in other groups, even though if it did not pass the significant *p* value threshold. Similarly, if a site was designated as hyper- (c) or hypo-methylated (d) in at least one group, similar pattern was most likely observed in other groups as well.

Supplementary Figure 8. Map of transcripts from paralogous genes, showing syntenic regions of the Atlantic salmon genome, based on the expressed sequences.

Supplementary Figure 9. Log 2 fold change differences in expression profiles of duplicated genes where both copies have significantly changed their levels of transcript abundances in response to stress.

Supplementary Figure 10. Examples of comparative expression profiles of duplicated genes in the control (*CO*) and across the stressed treatment groups (embryonic (*E*), post-hatch (*PH*) and embryonic and post-hatch (*EPH*)). Blue and the red dots represent the expression for either of the gene paralogues.

Supplementary Figure 11. Pie charts, showing the numbers and percentages of differentially hyper- or hypo-methylated sites across the treatment groups (embryonic (*E*), post-hatch (*PH*) and embryonic and post-hatch (*EPH*)).

Supplementary Figure 12. Percentage of methylated Cs in the CpG context throughout the genomes of the replicates within the stressed groups, embryonic (*E*), post-hatch (*PH*) and embryonic and post-hatch (*EPH*) as well as the control individuals (*CO*).

Supplementary Figure 13. Graphs, showing association between genes with significantly higher or lower levels of expression (box plots) with the average methylation level (red circles) in the three stressed groups, embryonic (*E*), post-hatch (*PH*) and embryonic and post-hatch (*EPH*).

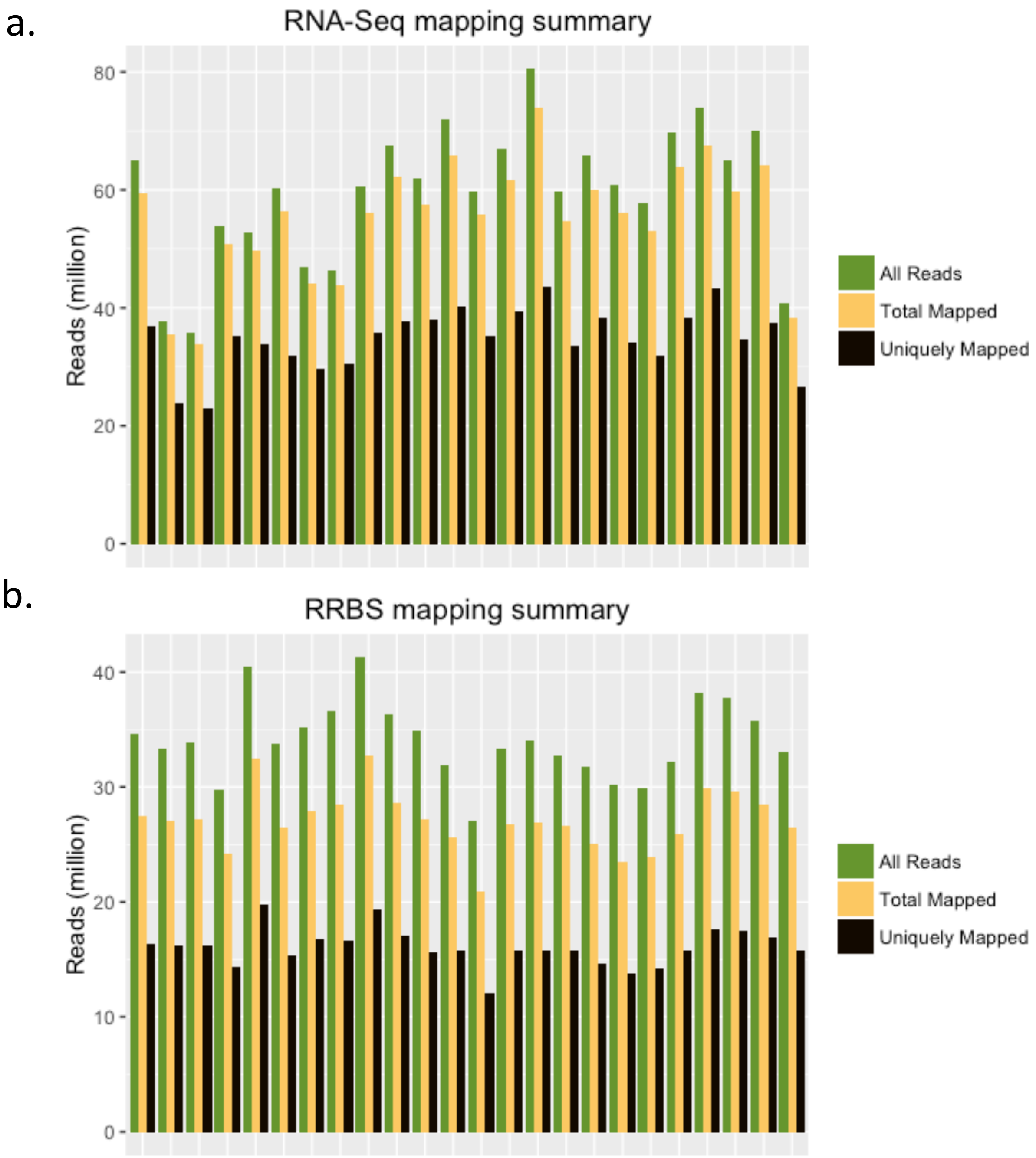

Supplementary Figure 1.

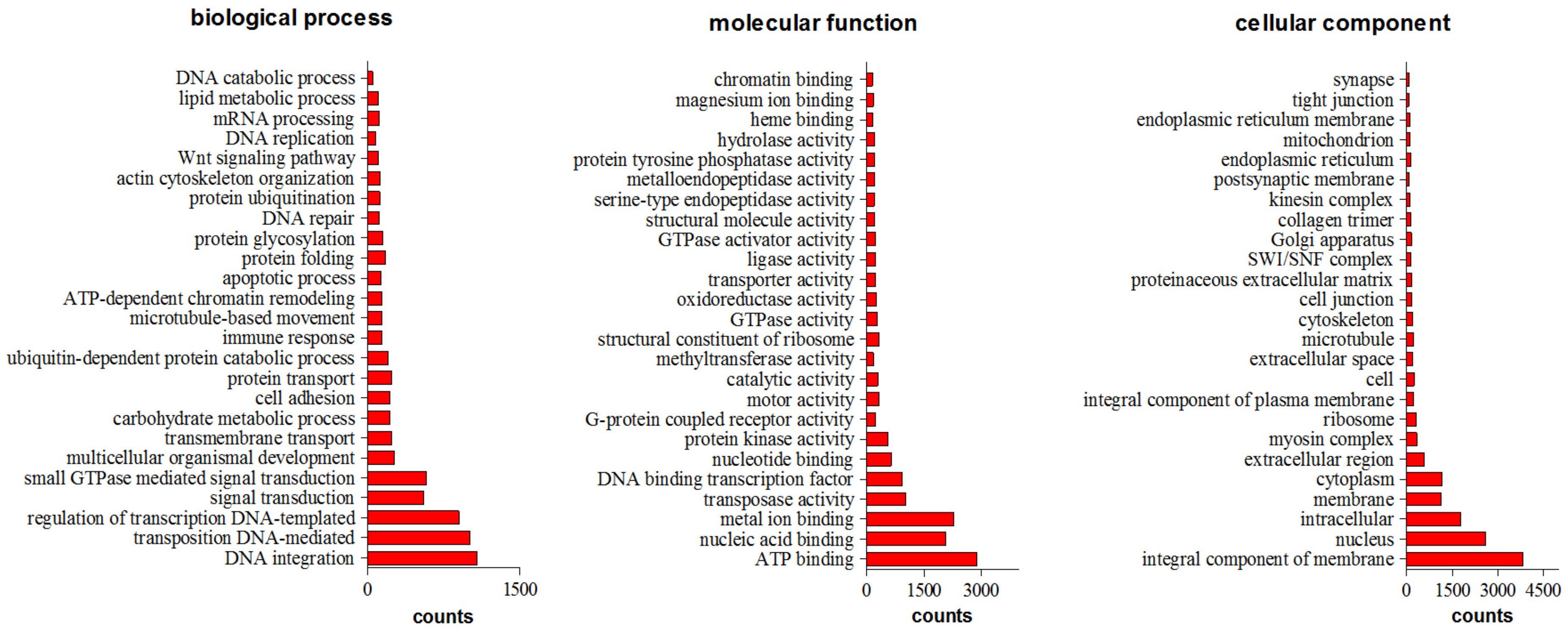

Supplementary Figure 2.

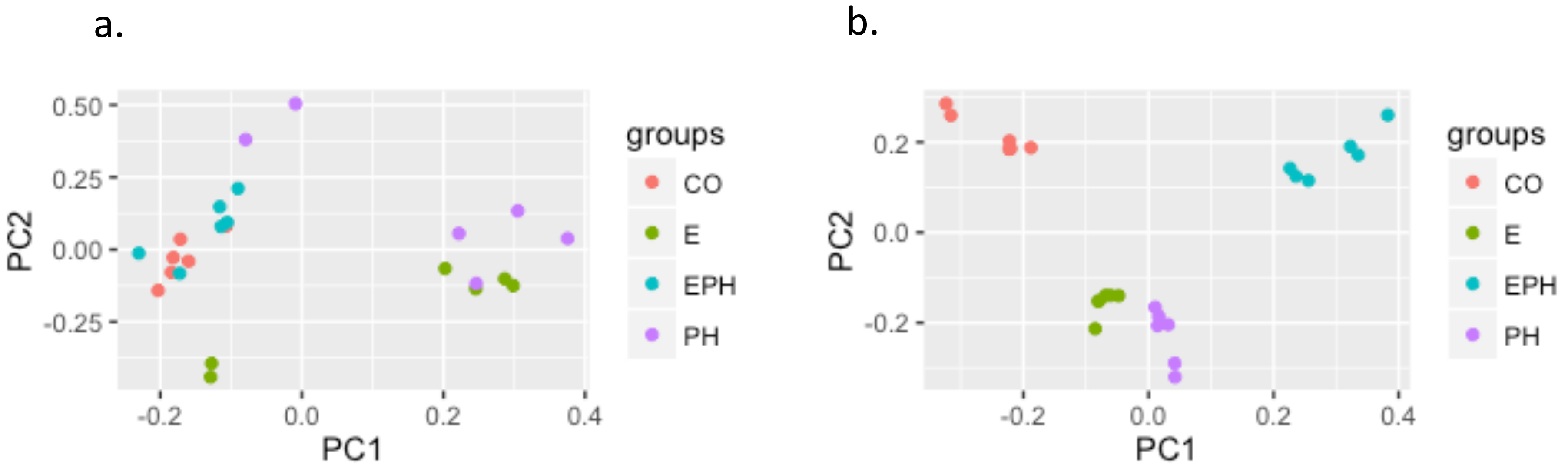

Supplementary Figure 3.

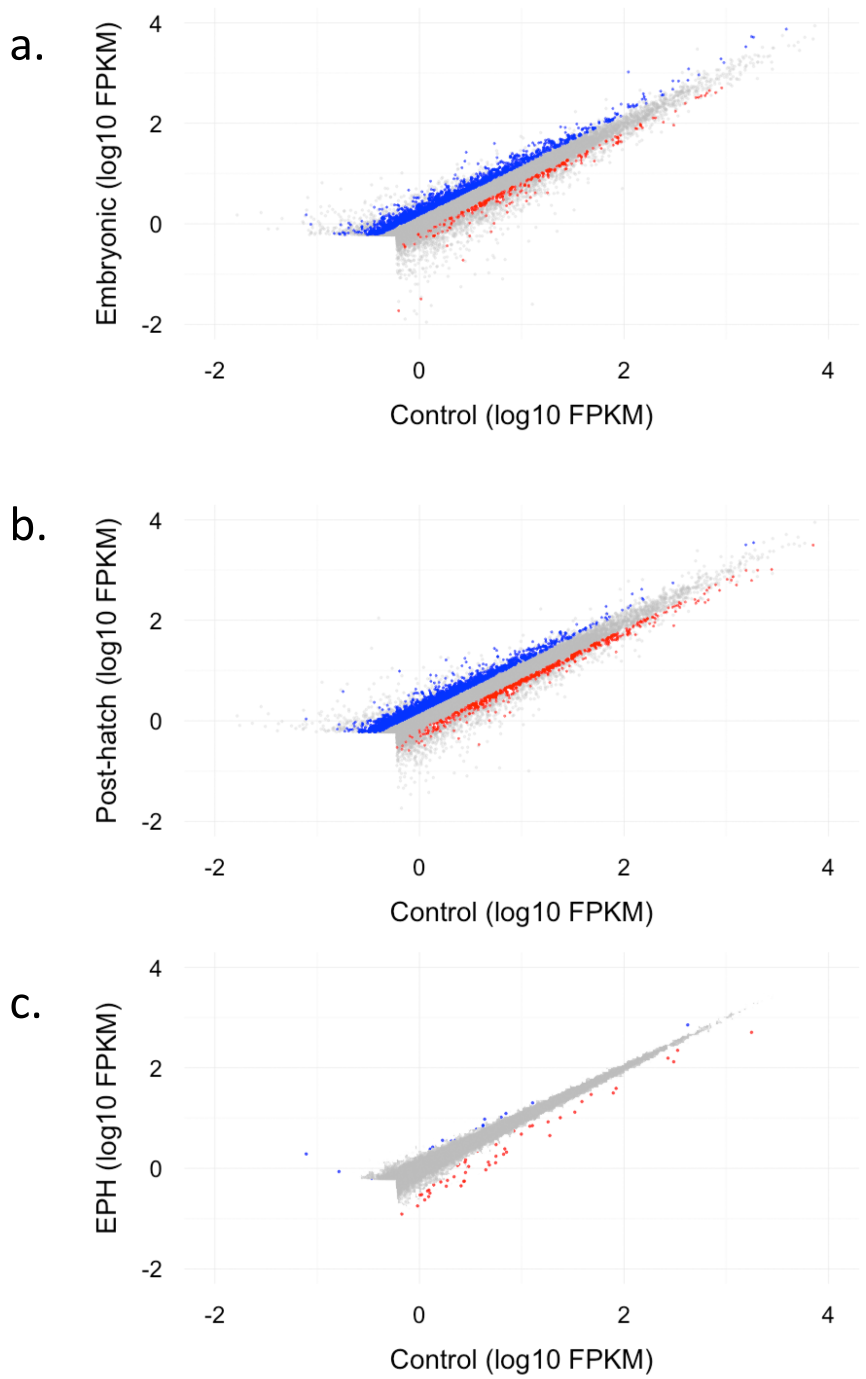

Supplementary Figure 4.

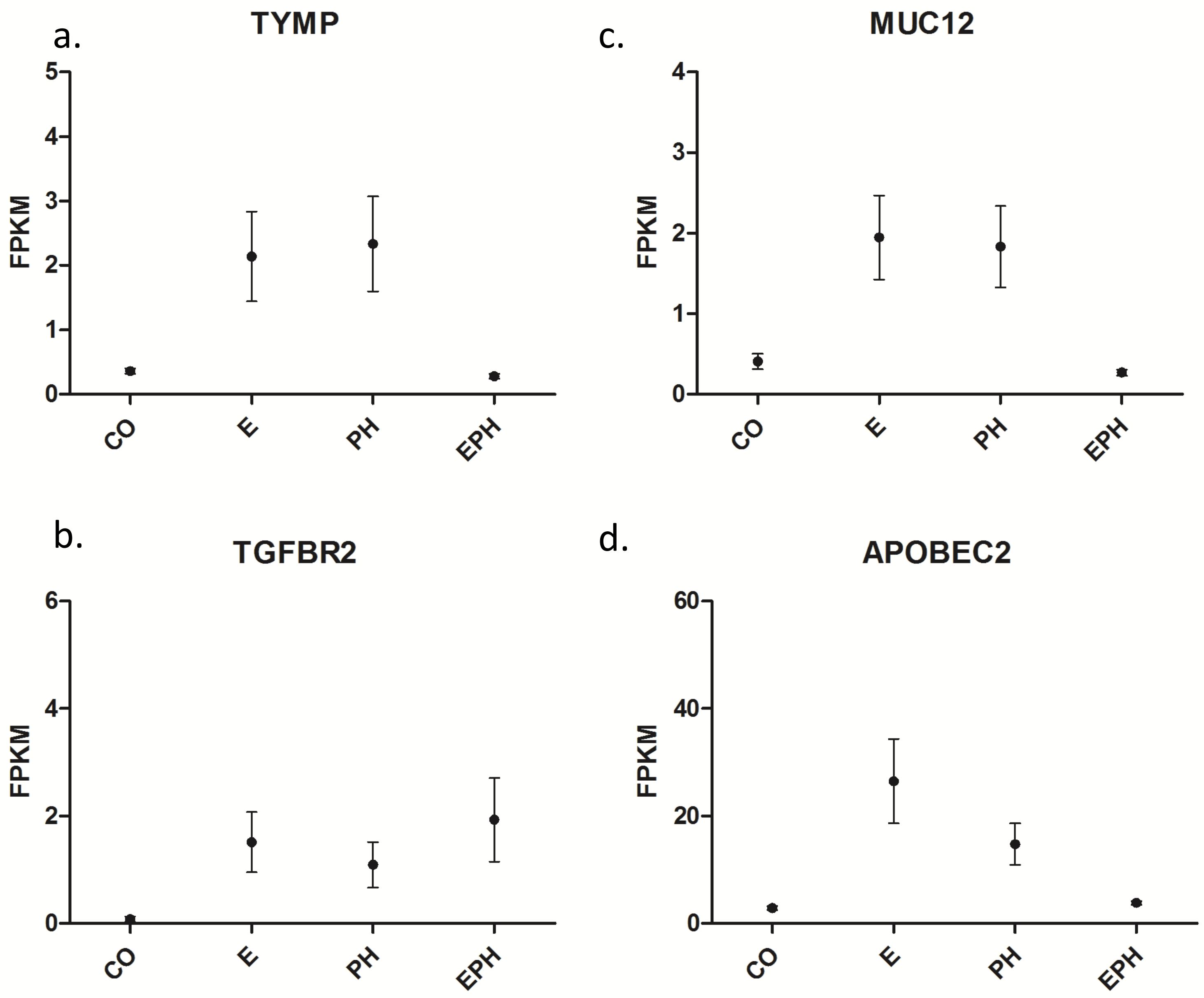

Supplementary Figure 5.

a.

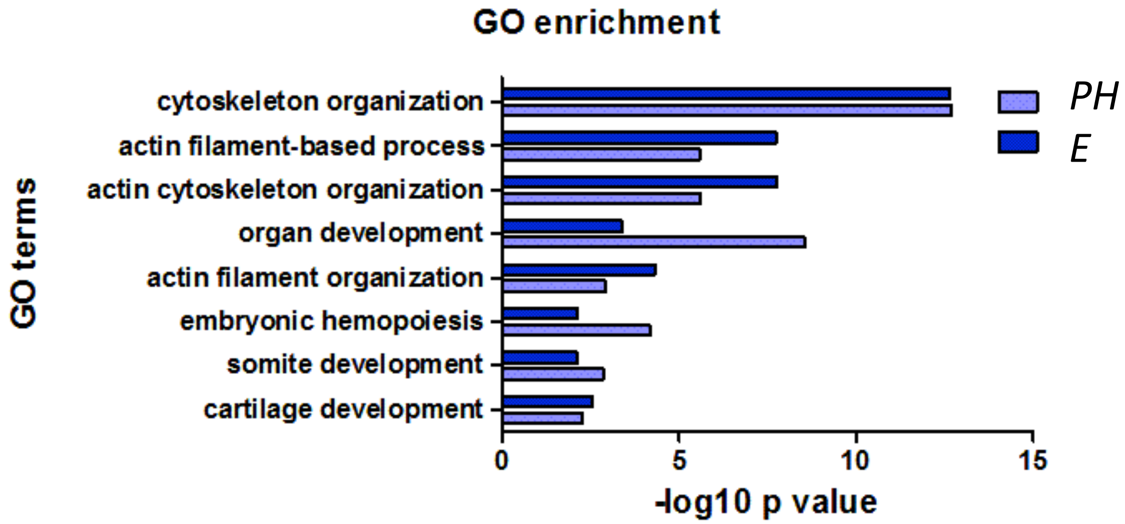

b.

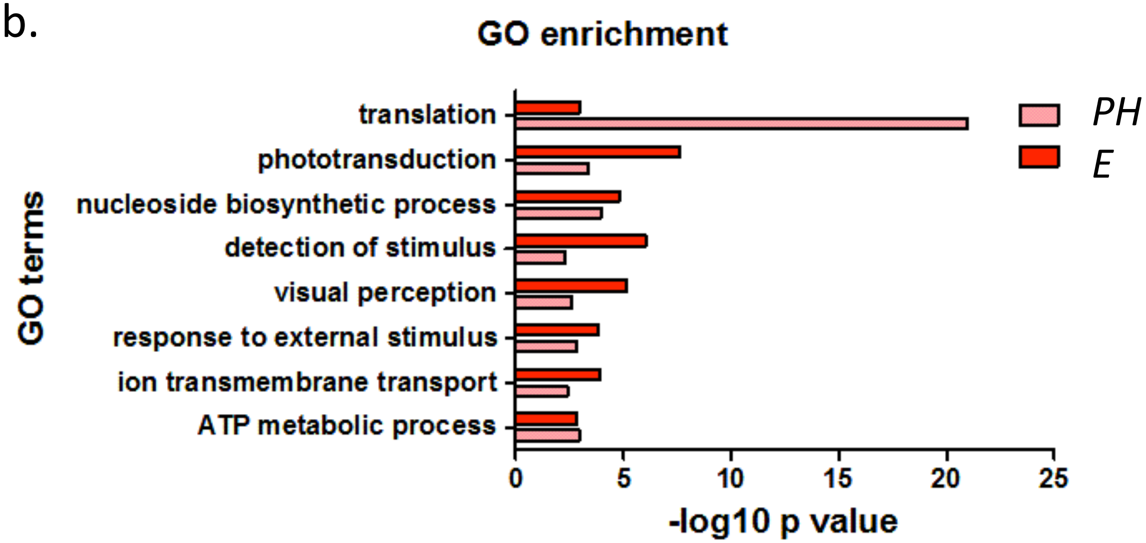

Supplementary Figure 6.

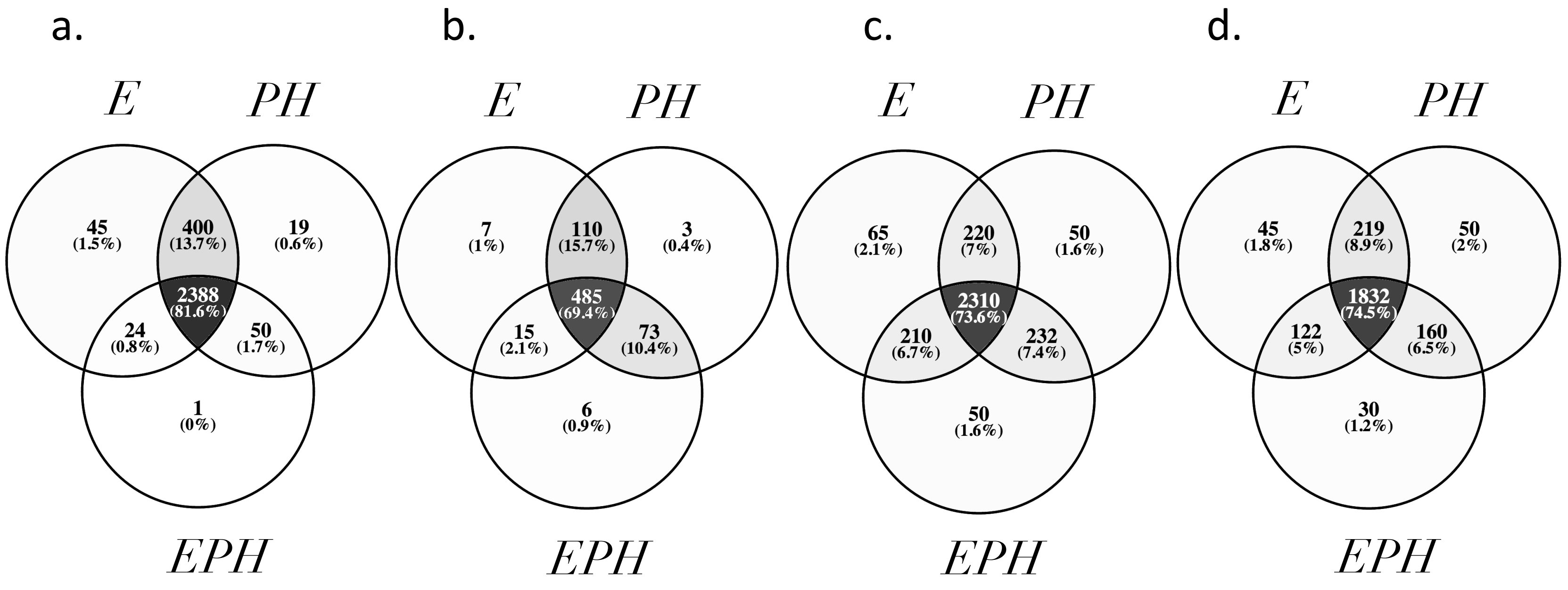

Supplementary Figure 7.

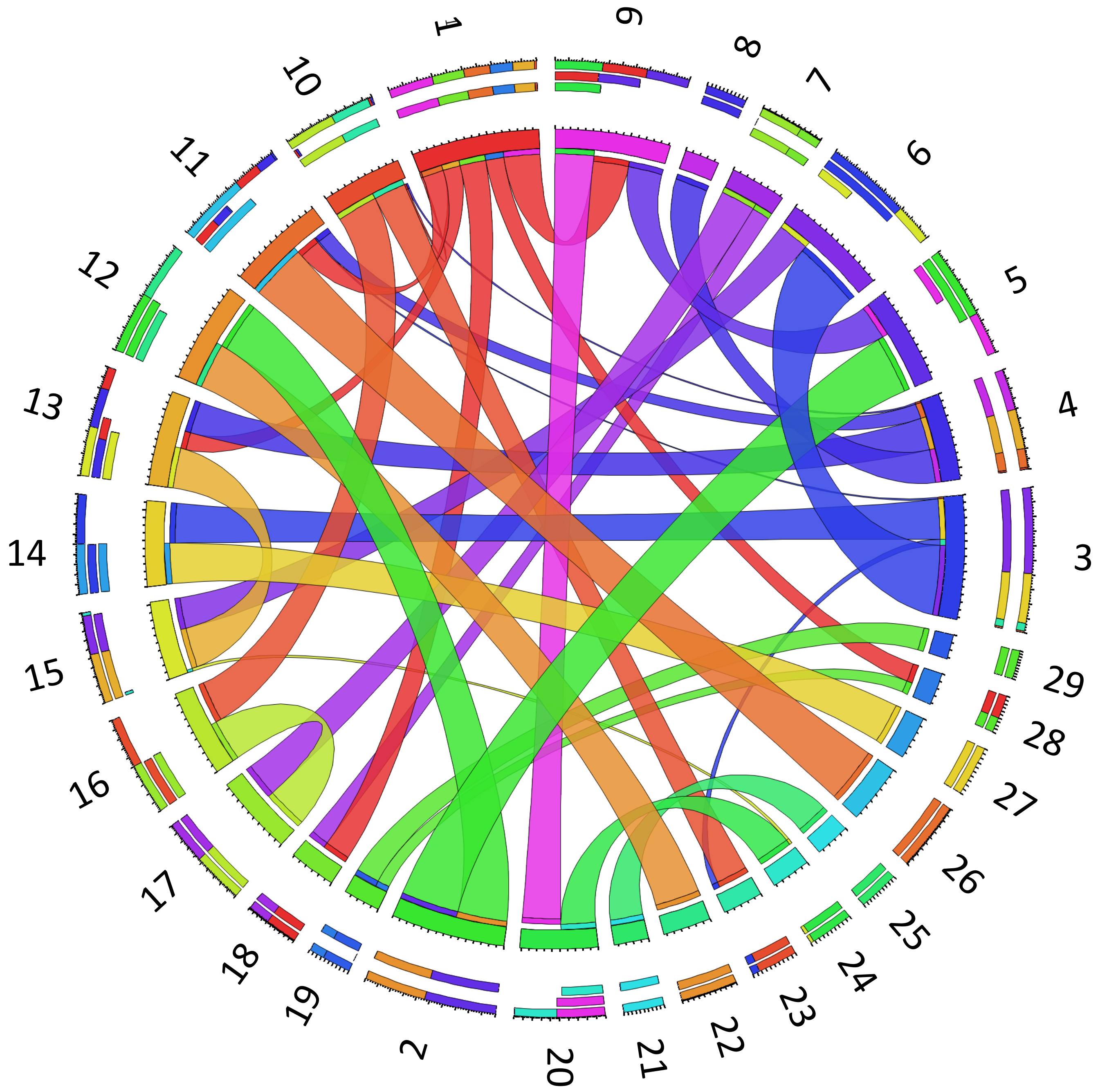

Supplementary Figure 8.

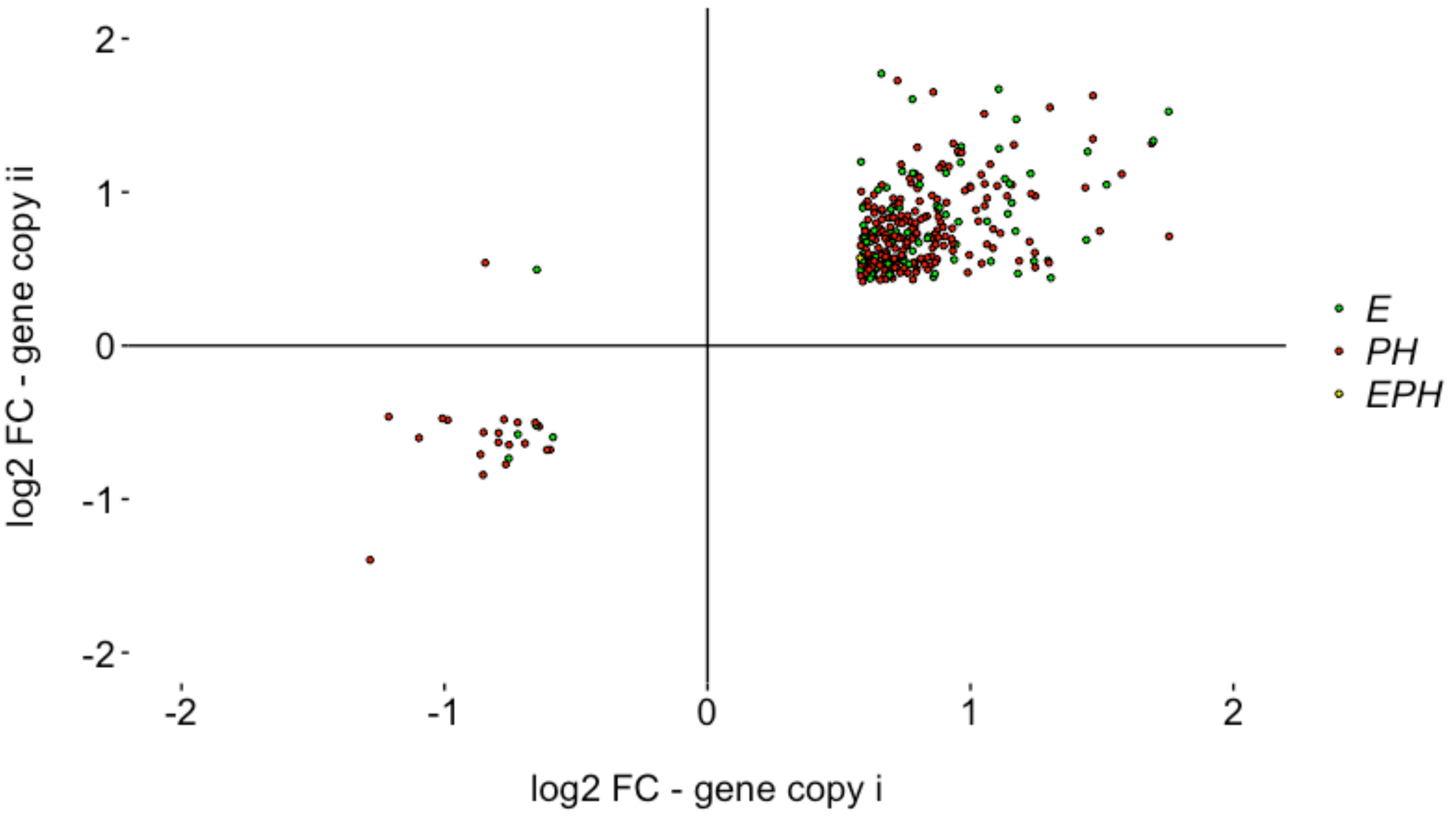

Supplementary Figure 9.

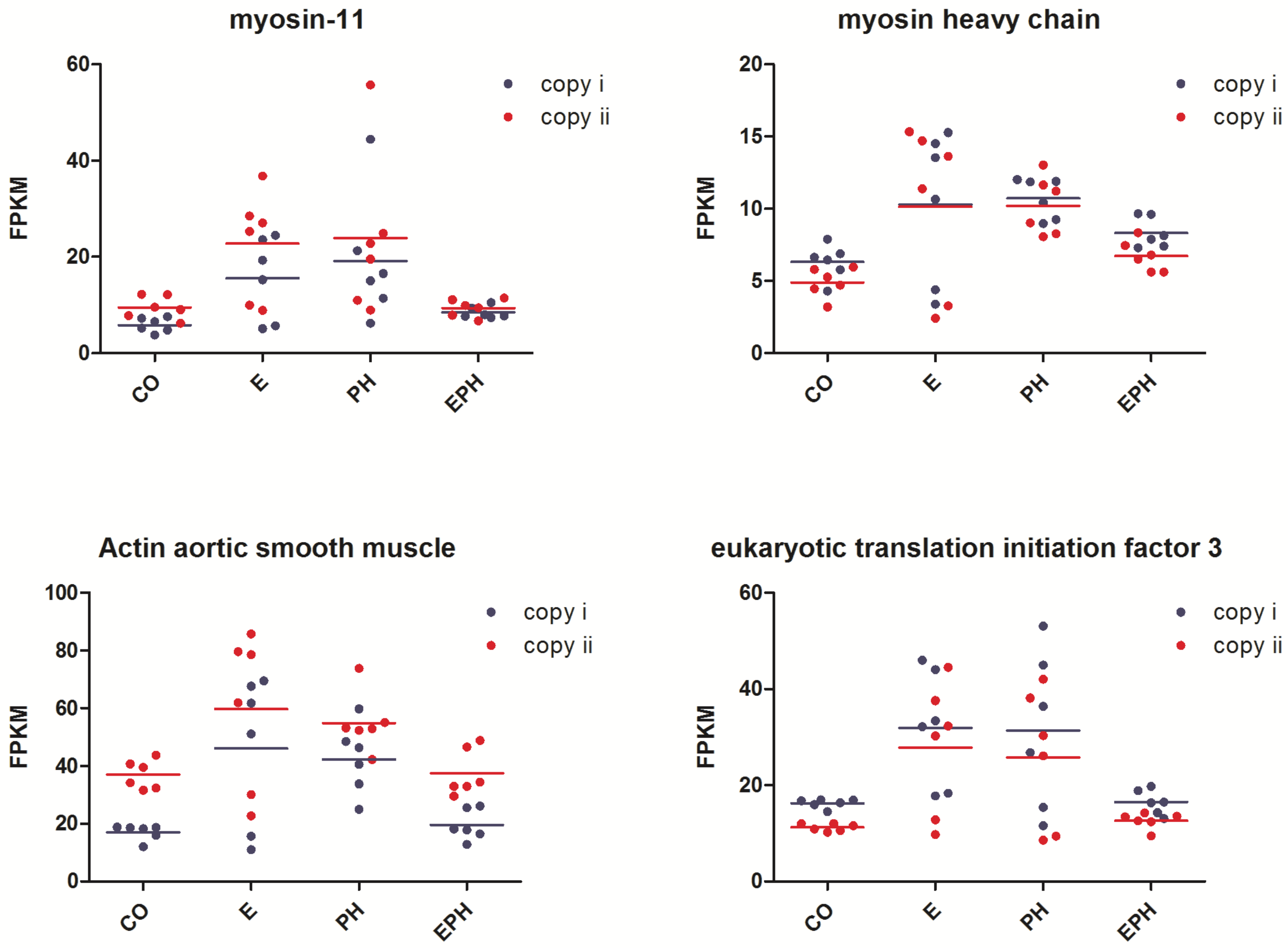

Supplementary Figure 10.

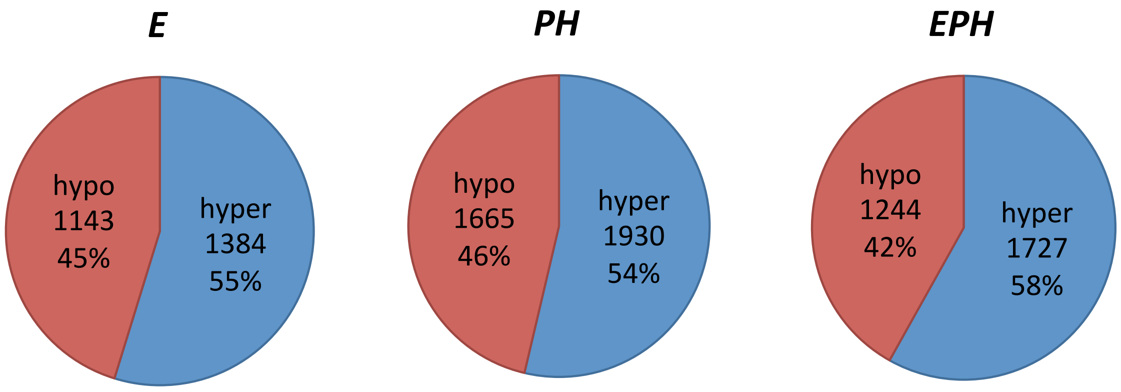

Supplementary Figure 11.

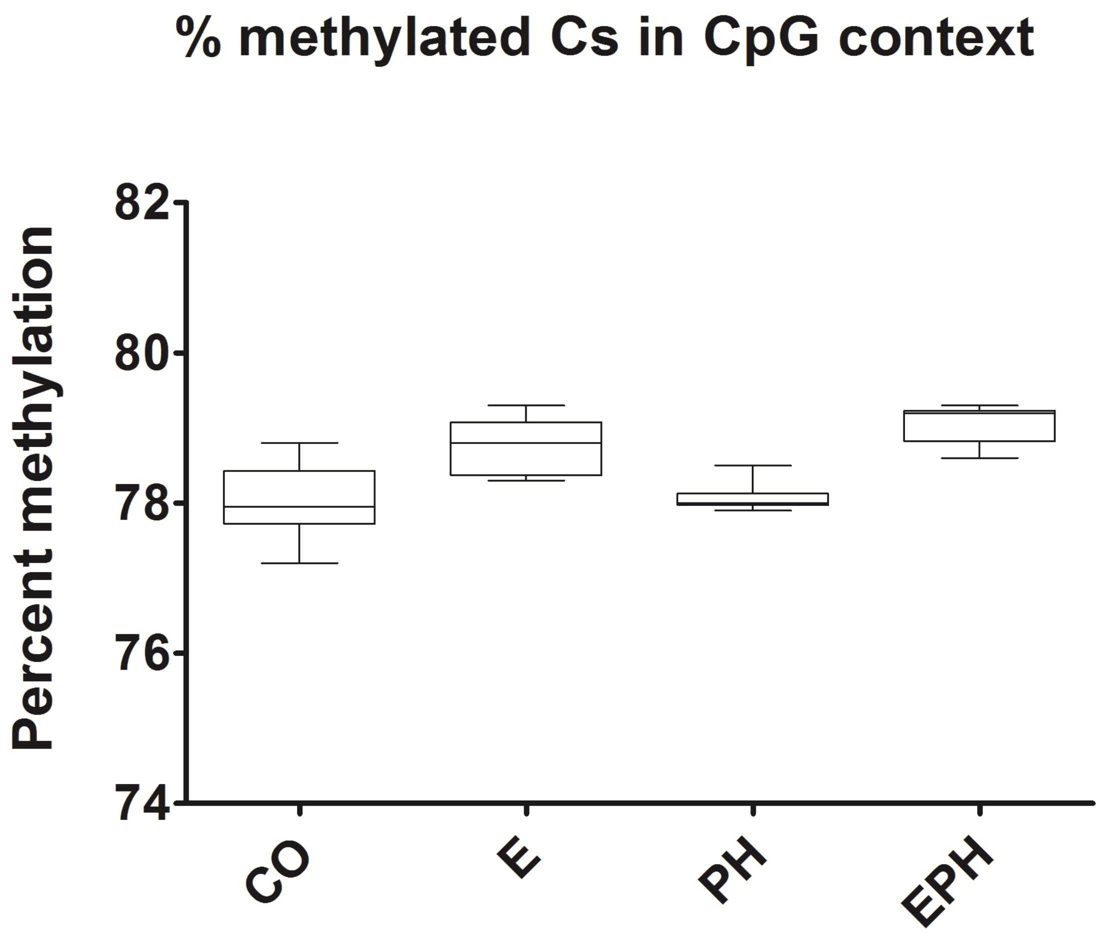

Supplementary Figure 12.

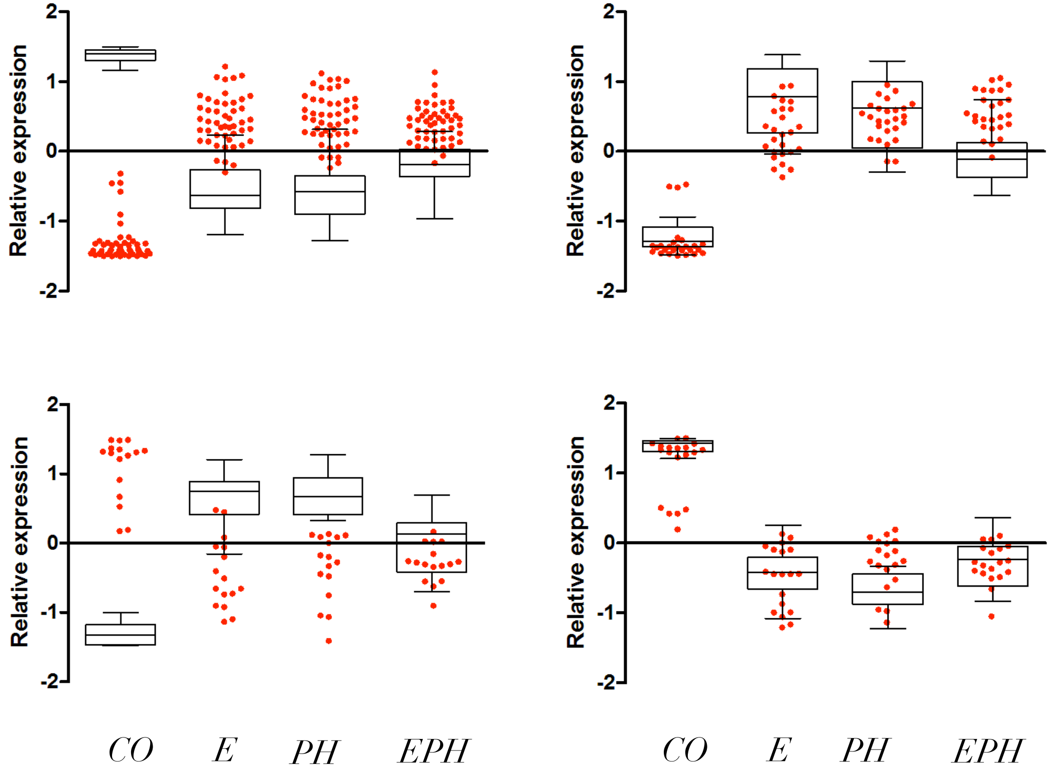

Supplementary Figure 13.

Supplementary Table 1. Association between relative levels of expression with relative changes in DNA methylation

						Relative gene expression				Relative average methylation			
	**gene name**	**symbol**	**chr**	**start**	**end**	***CO***	***E***	***PH***	***EPH***	***CO***	***E***	***PH***	***EPH***
1	Intracellular hyaluronan-binding protein 4	Habp4	ssa01	129529531	129537336	1.42	-0.27	-0.92	-0.23	1.35	0.07	-0.98	-0.44
2	**Collagen alpha-1 chain-like**	**Col1a1**	ssa03	7701163	7744424	1.43	-0.06	-0.69	-0.69	1.23	-1.22	0.08	-0.09
3	**arginine methyltransferase 1**	**PRMT1**	ssa03	50278208	50284748	1.25	-0.51	-1.04	0.30	1.29	-0.42	0.18	-1.06
4	kyphoscoliosis peptidase	**Ky**	ssa05	19721981	19732264	1.38	-0.89	-0.54	0.06	0.50	-0.10	-0.32	-0.08
5	RING-box protein 1	**Rbx1**	ssa05	25217863	25218336	1.43	-0.42	-0.87	-0.14	1.30	-0.10	-1.14	-0.05
6	**growth factor receptor-bound protein 2**	**Grb2**	ssa06	29167836	29211829	1.21	0.12	-1.23	-0.10	0.48	0.13	-0.27	-0.33
7	Eukaryotic translation initiation factor 5-like protein	**Eif5**	ssa09	26173570	26219391	1.45	-0.22	-0.40	-0.83	1.42	-0.74	-0.01	-0.66
8	casein kinase 1, alpha 1	**Csnk1a1**	ssa09	71494732	71520172	1.21	-1.08	-0.49	0.36	1.26	-1.17	-0.18	0.10
9	Proteasome subunit alpha type 4	**Psma4**	ssa10	62938362	62938660	1.48	-0.43	-0.70	-0.36	1.33	-0.45	-0.39	-0.50
10	synaptoporin-like	**Synpr**	ssa12	49641164	49659163	1.44	-0.32	-0.88	-0.23	1.49	-0.45	-0.64	-0.40
11	striping, autosomal 3	**Sta3**	ssa13	21640246	21650780	1.46	-0.40	-0.80	-0.26	1.38	-1.00	-0.11	-0.28
12	prolyl 4-hydroxylase subunit alpha-2-like	**P4ha2**	ssa13	54365107	54368577	1.49	-0.44	-0.44	-0.62	0.42	0.00	-0.27	-0.15
13	zinc finger and SCAN domain-containing 2	**Zscan2**	ssa14	33845886	34062905	1.28	-1.07	-0.42	0.21	0.42	-0.13	-0.33	0.04
14	**BOLA class I histocompatibility antigen, alpha chain BL3-7-l**	**H2-Ab_pr1**	ssa14	50982878	51050371	1.45	-0.64	-0.70	-0.11	1.37	-0.45	-0.96	0.05
15	NADH dehydrogenase [ubiquinone] 1 alpha subcomplex	**Ndufa1**	ssa21	48498564	48508549	1.49	-0.66	-0.33	-0.49	1.33	-1.07	0.02	-0.28
16	C-C motif chemokine 19-like	**Ccl19**	ssa24	40996068	41020301	1.34	-0.20	-1.08	-0.06	1.42	-0.88	-0.12	-0.42
17	Proteasome subunit beta type-2	**Psmb2**	ssa27	7633357	7648173	1.31	0.25	-0.78	-0.79	1.50	-0.45	-0.53	-0.51
18	carboxymethylenebutenolidase homolog	**Cmbl**	ssa29	31337438	31338141	1.48	-0.73	-0.41	-0.34	1.36	-1.00	0.02	-0.38
19	protein FAM84A	Fam84a	jcf1000045124_0_0	4113	18159	1.40	-0.57	0.03	-0.85	-1.41	0.35	0.93	0.13
20	ribonuclease T2-like	Rnaset2a	ssa01	93906958	93919152	1.41	-0.83	-0.58	0.00	-1.46	0.69	0.60	0.18
21	ABC transporter G family member 20-like	Abcg2	ssa01	116570338	116672140	1.41	-0.86	-0.05	-0.51	-0.46	0.26	-0.09	0.28
22	vacuolar protein sorting-associated protein	Vps37b	ssa01	137763780	137795927	1.50	-0.44	-0.51	-0.54	-1.23	0.33	-0.23	1.14
23	tropomyosin beta chain	Tpm2	ssa01	150170633	150207465	1.27	-0.93	0.31	-0.65	-1.47	0.31	0.76	0.41
24	uncharacterized	-	ssa02	6973264	6975041	1.36	-0.05	-1.04	-0.26	-1.32	1.06	0.23	0.03
25	Esox lucius M-protein, striated muscle	Myom1	ssa03	330727	339995	1.32	0.23	-0.76	-0.80	-1.28	-0.13	1.12	0.29
26	endoplasmic reticulum-Golgi intermediate	Ergic1	ssa03	47891733	47891955	1.42	-0.07	-0.51	-0.84	-1.49	0.41	0.64	0.44
27	ubiquitin carboxyl-terminal hydrolase	Uchl1	ssa04	56503458	56538048	1.44	-0.24	-0.87	-0.34	-1.48	0.30	0.48	0.70
28	double C2-like domain-containing protein	Doc2a	ssa04	63884428	64069399	1.32	-0.45	-1.02	0.15	-0.58	-0.20	0.26	0.52
29	high-mobility group box 1 transcript	Hbp1	ssa04	79505654	79520840	1.32	-1.09	-0.24	0.01	-1.23	1.22	0.07	-0.06
30	**methyltransferase-like protein 25**	**Mettl25**	ssa07	50918914	51325550	1.47	-0.23	-0.58	-0.66	-0.32	0.36	-0.09	0.04
31	succinyl-CoA ligase subunit alpha, mitochondrial-like	Suclg1	ssa08	5912552	5945000	1.44	-0.68	-0.68	-0.09	-1.31	1.04	-0.16	0.43
32	**actin, alpha cardiac-like**	**Actc1**	ssa09	20065647	20070085	1.39	-0.92	-0.46	-0.02	-1.41	0.84	0.52	0.06
33	toxin CdiA-like transcript	-	ssa09	48189043	48225917	1.47	-0.72	-0.48	-0.27	-1.43	0.76	0.09	0.57
34	**guanidinoacetate N-methyltransferase**	**Gamt**	ssa10	15529022	15533796	1.25	-1.19	-0.04	-0.02	-1.49	0.46	0.42	0.62
35	60S ribosomal protein L13	Rpl13	ssa11	18891895	18897485	1.48	-0.69	-0.44	-0.35	-1.48	0.71	0.32	0.45
36	serine/threonine-protein phosphatase 6	Ppp6c	ssa11	44517534	44553878	1.30	-0.74	-0.83	0.27	-1.33	1.09	0.05	0.20
37	Eukaryotic translation initiation factor	Eif6	ssa12	31912723	31919433	1.24	-1.08	-0.45	0.28	-1.47	0.75	0.25	0.47
38	adaptin ear-binding coat-associated protein	Necap2	ssa12	60267506	60281508	1.34	-0.74	-0.78	0.19	-1.44	0.57	0.69	0.18
39	**receptor-type tyrosine-protein phosphatase gamma isoform**	**Ptprg**	ssa12	66123841	66452946	1.26	-1.15	-0.26	0.15	-0.90	0.06	0.26	0.58
40	5'-nucleotidase domain-containing protein	Nt5dc1	ssa13	26413795	26457072	1.30	-0.61	-0.93	0.24	-1.41	0.23	0.80	0.38

41	Leucyl-tRNA synthetase, cytoplasmic	Lars	ssa13	51859612	51886023	1.27	0.10	-1.15	-0.22	-1.35	0.07	0.98	0.30
42	calpain small subunit 1	Capns1	ssa13	62001860	62012337	1.27	-0.14	-1.17	0.04	-1.28	-0.30	0.91	0.67
43	STARD3 N-terminal like transcript	Stard3	ssa14	3617802	3670177	1.43	-0.70	-0.67	-0.06	-1.50	0.42	0.60	0.48
44	lipoprotein lipase-like	Lpl	ssa14	41713246	41726768	1.49	-0.62	-0.33	-0.54	-1.46	0.80	0.38	0.28
45	**tyrosine phosphatase type IVA 2**	**Ptp4a2**	ssa14	81621566	81675938	1.45	-0.83	-0.37	-0.25	-0.45	0.09	0.28	0.08
46	tribbles homolog 2-like	Trib1	ssa15	49154380	49164705	1.35	-0.61	-0.89	0.16	-1.48	0.47	0.30	0.71
47	**pancreatic progenitor cell differentiation**	**Ppdpf**	ssa15	98601168	98604631	1.37	-0.59	-0.88	0.10	-1.46	0.81	0.39	0.26
48	cytochrome b-c1 complex subunit 6, mitochondrial-like	Uqcrh	ssa16	47752094	47759607	1.48	-0.63	-0.57	-0.28	-1.32	1.07	-0.08	0.33
49	uncharacterized	-	ssa16	76735118	76748097	1.41	-0.21	-0.23	-0.96	-1.50	0.47	0.54	0.49
50	WW domain binding protein 1-like	Wbp11	ssa18	13013426	13071980	1.41	-0.94	-0.15	-0.33	-1.31	0.43	1.04	-0.16
51	GTP-binding protein SAR1b-like	Sar1b	ssa18	27311776	27342819	1.44	-0.78	-0.55	-0.11	-1.44	0.70	0.55	0.19
52	protein kinase C and casein kinase substrate	Pacsin1	ssa19	53019527	53035703	1.48	-0.39	-0.72	-0.36	-1.36	0.09	0.74	0.54
53	**actin, beta**	**Actb**	ssa19	57284794	57297525	1.16	-0.01	-1.28	0.13	-1.42	0.35	0.92	0.16
54	interleukin-15 receptor subunit alpha isoform X2	Il15ra	ssa20	42524202	42525019	1.46	-0.70	-0.58	-0.18	-1.33	-0.15	0.53	0.96
55	cytochrome P450 2M1	Cyp2b10	ssa20	46190063	46280054	1.47	-0.36	-0.35	-0.76	-1.50	0.58	0.44	0.48
56	interferon alpha/beta receptor 1a-like	Ifnar1	ssa21	17606996	17607189	1.48	-0.68	-0.48	-0.32	-1.46	0.15	0.69	0.62
57	acyl-coenzyme A-binding protein	Acbd3	ssa21	30946862	30951767	1.31	-0.62	-0.92	0.23	-1.40	0.15	0.73	0.51
58	transketolase-like	Tktl1	ssa22	42095501	42100661	1.27	-1.14	0.14	-0.26	-1.48	0.63	0.33	0.52
59	lysosome-related organelle complex-1 subunit 1	Bloc1s5	ssa22	53041391	53053924	1.46	-0.70	-0.57	-0.19	-1.47	0.51	0.25	0.71
60	claudin-4-like	Cldn4	ssa24	14005889	14007823	1.28	-0.09	-1.16	-0.03	-1.44	0.15	0.48	0.81
61	dachshund homolog 1-like	Dach1	ssa25	2538286	2811537	1.36	-0.25	-1.04	-0.08	-1.03	0.36	0.29	0.38
62	**Death-associated protein-like 1-B**	**Dapl1**	ssa25	12134339	12270539	1.42	-0.41	-0.90	-0.10	-1.43	0.31	0.75	0.37
63	**serine/threonine-protein phosphatase 2A**	**Ppp2r1a**	ssa26	2655915	2660501	1.28	-0.87	0.31	-0.71	-1.42	0.62	0.10	0.70
64	uncharacterized	-	ssa27	14332800	14335568	1.33	-0.76	-0.78	0.20	-1.34	0.24	1.03	0.07
65	neurogranin, TIP41-like protein MHC class	Nrgn	ssa28	15553537	15570989	1.41	-0.31	-0.95	-0.14	-1.50	0.59	0.46	0.45
66	LSM12 homolog	Lsm12	ssa28	18769611	18781970	1.29	0.16	-1.09	-0.36	-1.31	0.17	1.01	0.13
67	Uncharacterized protein	-	ssa01	23342207	23344359	-1.01	0.77	0.94	-0.71	1.32	-0.65	-0.33	-0.34
68	cilia and flagella associated protein 46	Cfap46	ssa01	51389328	51411819	-1.27	-0.15	1.14	0.28	1.47	-0.65	-0.20	-0.62
69	Zymogen granule membrane protein 16	Zg16	ssa11	38964121	39283096	-1.25	0.91	0.69	-0.35	0.17	0.08	-0.28	0.02
70	PH and SEC7 domain-containing protein	Psd	ssa11	64641886	64664632	-1.47	0.76	0.41	0.30	1.30	-0.40	-1.06	0.16
71	sodium-coupled neutral amino acid transporter 3-like	Slc38a3	ssa13	26524241	26563245	-1.17	-0.08	1.27	-0.02	1.25	-0.06	-1.04	-0.15
72	putative ubiquitin C variant 1	Ubc	ssa13	72691991	72694650	-1.48	0.57	0.63	0.28	0.91	0.47	-1.40	0.02
73	Rab interacting lysosomal protein-like 1	Rilpl1	ssa13	91446017	91564985	-1.48	0.49	0.71	0.28	1.48	-0.72	-0.47	-0.28
74	**syncoilin, intermediate filament protein**	**Sync**	ssa13	102051186	102088673	-1.32	0.39	1.05	-0.12	1.31	-1.09	0.09	-0.30
75	paralemmin 2	Palm2	ssa13	105791222	105850987	-1.01	1.20	0.41	-0.60	0.66	-0.50	-0.18	0.02
76	xin actin-binding repeat-containing protein 1-like	Xirp1	ssa14	49037808	49060350	-1.45	0.84	0.33	0.29	1.20	0.44	-0.75	-0.89
77	ribosomal protein L15	Rpl15	ssa14	68236871	68240471	-1.00	1.04	0.65	-0.69	1.28	-1.13	0.11	-0.27
78	CD82 antigen	Cd82	ssa16	1164640	1225217	-1.19	1.00	0.62	-0.44	1.34	-0.92	0.13	-0.55
79	inactive serine protease PAMR1-like	Pamr1	ssa16	20545023	20673139	-1.46	0.80	0.37	0.30	1.35	-0.90	0.09	-0.55
80	uncharacterized	-	ssa18	12374799	12557766	-1.38	-0.09	0.78	0.69	0.52	-0.20	0.00	-0.32
81	serine/arginine-rich splicing factor 10-like	Srsf10	ssa27	35231270	35239433	-1.34	0.56	0.93	-0.15	1.48	-0.73	-0.44	-0.30
82	**myosin-7-like**	**Myh7**	ssa29	19760446	19991647	-1.48	0.74	0.39	0.34	0.19	-0.05	0.12	-0.26
83	**actin alpha 2**	**Actn2**	ssa01	65036622	65053673	-1.29	1.11	0.28	-0.10	-1.37	-0.09	0.58	0.88
84	**serine/threonine-protein kinase LMTK2-like**	**Lmtk2**	ssa01	76303924	76325653	-1.27	0.94	0.64	-0.30	-1.24	-0.37	0.96	0.65

85	G-protein-signaling modulator 1-like	Gpsm1	ssa02	25194007	25198619	-1.10	1.32	-0.19	-0.03	-1.37	0.17	0.18	1.02
86	sarcalumenin	Srl	ssa02	56698958	56708327	-1.09	1.33	-0.01	-0.23	-1.35	0.61	-0.14	0.88
87	RB1-inducible coiled-coil 1	Rb1cc1	ssa03	30985226	31005260	-1.37	0.77	0.72	-0.12	-1.41	0.58	0.44	0.39
88	3-mercaptopyruvate sulfurtransferase-like	Mpst	ssa03	54465347	54496150	-1.37	1.02	0.06	0.28	-0.50	0.03	0.29	0.17
89	**protein phosphatase, Mg2+/Mn2+ dependent,**	**Ppm1n**	ssa04	44709854	44727277	-1.07	1.07	0.60	-0.59	-1.48	0.72	0.42	0.35
90	sperm associated antigen 9	Spag9	ssa06	19660835	19677777	-1.17	1.17	0.36	-0.36	-1.37	0.36	0.55	0.46
91	Myristoylated alanine-rich C-kinase substrate	Marcks	ssa06	54473986	54478009	-1.09	1.24	0.28	-0.44	-0.47	-0.01	0.33	0.15
92	piggyBac transposable element-derived protein 4-like	Pgbd1	ssa07	2010705	2068416	-1.39	0.44	0.93	0.03	-1.27	-0.26	0.47	1.05
93	Aspartyl beta-hydroxylase-like	Asph	ssa07	24279979	24318533	-1.00	1.38	-0.29	-0.09	-1.47	0.35	0.66	0.45
94	protein phosphatase 1, regulatory subunit 3A	Ppp1r3a	ssa07	34078838	34085415	-1.44	0.80	0.51	0.13	-1.36	0.94	0.50	-0.08
95	2-oxoisovalerate dehydrogenase subunit alpha	Bckdha	ssa09	138907872	138910160	-1.32	-0.03	1.08	0.27	-1.47	0.28	0.76	0.43
96	basigin	Bsg	ssa10	19104327	19118654	-0.98	0.23	1.30	-0.55	-1.42	0.93	0.16	0.33
97	transcription factor E2-alpha-like	Tcf3	ssa10	19978431	20018217	-1.06	0.66	1.02	-0.63	-1.49	0.49	0.50	0.51
98	NUAK family SNF1-like kinase 1	Nuak1	ssa10	73800291	73828765	-1.17	0.24	1.22	-0.28	-1.46	0.31	0.60	0.55
99	gamma-aminobutyric acid receptor subunit	Gabrd	ssa10	108198386	108204632	-1.34	0.14	1.09	0.11	-1.35	0.61	-0.14	0.88
100	unnamed protein product	-	ssa11	1341935	1368520	-1.49	0.42	0.69	0.38	-1.41	-0.01	0.68	0.74
101	Ribosomal protein L23a	Rpl23a	ssa11	93010004	93016976	-0.97	1.37	0.01	-0.42	-1.44	0.08	0.62	0.74
102	**Myosin heavy chain**	**Myh1**	ssa12	4675370	4855042	-1.23	1.15	0.34	-0.26	-1.46	0.74	0.36	0.36
103	uncharacterized	-	ssa13	43558913	43559400	-1.40	-0.03	0.71	0.73	-1.41	0.10	0.83	0.49
104	nesprin-2-like isoform X8	Syne2	ssa15	39177157	39178312	-1.31	0.10	1.13	0.09	-1.30	-0.26	0.87	0.69
105	uncharacterized	-	ssa15	95121054	95354538	-1.32	0.62	0.91	-0.22	-0.51	0.24	0.16	0.11
106	ATP-binding cassette, sub-family B	Abcb10	ssa17	4270332	4313057	-1.39	0.27	1.00	0.12	-1.35	-0.04	0.42	0.96
107	collagen alpha-1 chain-like	Col22a1	ssa28	26795699	26819355	-1.36	0.76	-0.14	0.74	-1.41	0.79	0.10	0.52
108	phosphate regulating endopeptidase homolog	Phex	ssa29	41847222	41847668	-0.94	1.39	-0.01	-0.44	-1.33	-0.18	0.61	0.90
